# Specific Posture-Stabilising Effects of Vision and Touch Are Revealed by Distinct Changes of Body Oscillation Frequencies

**DOI:** 10.3389/fneur.2021.756984

**Published:** 2021-11-22

**Authors:** Stefania Sozzi, Antonio Nardone, Marco Schieppati

**Affiliations:** ^1^Centro Studi Attività Motorie (CSAM), Istituti Clinici Scientifici Maugeri SB (Istituto di Ricovero e Cura a Carattere Scientifico, IRCCS), Pavia, Italy; ^2^Neurorehabilitation and Spinal Unit, Department of Clinical-Surgical, Diagnostic and Pediatric Sciences, Istituti Clinici Scientifici Maugeri SB (Istituto di Ricovero e Cura a Carattere Scientifico, IRCCS), University of Pavia, Pavia, Italy; ^3^Istituti Clinici Scientifici Maugeri SB, Istituto di Ricovero e Cura a Carattere Scientifico (IRCCS), Pavia, Italy

**Keywords:** stance, critical conditions, body oscillation, spectral analysis, centre of foot pressure, length and area of sway path, vision, haptic

## Abstract

We addressed postural instability during stance with eyes closed (EC) on a compliant surface in healthy young people. Spectral analysis of the centre of foot pressure oscillations was used to identify the effects of haptic information (light-touch, EC-LT), or vision (eyes open, EO), or both (EO-LT). Spectral median frequency was strongly reduced by EO and EO-LT, while spectral amplitude was reduced by all “stabilising” sensory conditions. Reduction in spectrum level by EO mainly appeared in the high-frequency range. Reduction by LT was much larger than that induced by the vision in the low-frequency range, less so in the high-frequency range. Touch and vision together produced a fall in spectral amplitude across all windows, more so in anteroposterior (AP) direction. Lowermost frequencies contributed poorly to geometric measures (sway path and area) for all sensory conditions. The same subjects participated in control experiments on a solid base of support. Median frequency and amplitude of the spectrum and geometric measures were largely smaller when standing on solid than on foam base but poorly affected by the sensory conditions. Frequency analysis but not geometric measures allowed to disclose unique tuning of the postural control mode by haptic and visual information. During standing on foam, the vision did not reduce low-frequency oscillations, while touch diminished the entire spectrum, except for the medium-high frequencies, as if sway reduction by touch would rely on rapid balance corrections. The combination of frequency analysis with sensory conditions is a promising approach to explore altered postural mechanisms and prospective interventions in subjects with central or peripheral nervous system disorders.

## Introduction

The sensory control of bipedal human stance has been a matter of investigation for many years (1–3). A plethora of studies has been published on this topic, including some from our group (4, 5). Body sway when standing upright on a solid base of support is normally almost negligible in healthy subjects, witnessing accurate and precise neural control (6, 7) based on the internal model of gravitational and inertial forces (8) and on multiple inputs from the receptors detecting the body state. The excursions of the centre of foot pressure (CoP) of subjects standing quietly on the firm ground are approximately contained within the size of a dime, even if there is a large variability in sway across different healthy subjects (9). In several conditions, though, sway area can significantly increase, such as standing on sloped surfaces or when leaning forward or backward (4, 10), or decrease when subjects stand on elevated platforms (11). Standing on viscoelastic, compliant support like a foam pad produces larger sway and obvious body unsteadiness (12–14). This can in some cases lead to falls (15, 16), especially when vision is not available (17) or when sensory deficits are present (18–20).

Vision is important for body stabilisation during standing (21, 22). Sway may increase without vision compared to eyes open during quiet stance on a firm platform (23) with the effects depending on the distance between the feet (22, 24–26). Vision is also able to gate the effects of vibration (activating the primary receptors of the muscle spindles) of the neck muscles, consisting of a large forward sway when the eyes are closed (27). Vision also moderates the postural effects of the Achilles tendon vibration (28) and plays a more important role in postural stability under challenging conditions compared to quiet stances, such as on a mobile platform or on a foam surface (13, 29–31). When vision is available, subjects reduce reliance on proprioception and increase reliance on visual information (25).

Proprioception is crucial in the control of body stability and orientation in space (32–35), and various manoeuvres have been put in place to clarify its role, including muscle vibration as a tool for activating the spindles (36) or leg ischemia by compression to attenuate the transmission of their firing (37, 38). However, the contribution of the spindles to standing posture may not have been completely elucidated, not to speak of the role of the information from the foot sole and from the intrinsic foot muscles (39–41). These inputs under a quiet stance on the firm ground would play a limited function because the information originating in the primary spindle terminations, which are mainly sensitive to the velocity of muscle stretch (42), may not be crucial in the absence of rapid changes in muscle length. Under a quiet stance, the small-diameter fibres originating in the secondary spindle terminations may play a predominant role (43). Further, reweighting of the proprioceptive information normally occurs, as attested by the reduction in the amplitude of the soleus muscle H-reflex during unperturbed stance (44). Moreover, the reflex excitability of the motor neurons of the leg muscles is decreased when the stance is stabilised by holding onto a solid frame (45, 46) or by lightly touching fixed support (47). The role of proprioception can be more important when the balance is challenged (48) without vision. Under perturbed conditions or with a major reduction of the support surface (49, 50), when the postural muscle activity plays a major stabilising role, the role of proprioception is amplified and that of vision becomes of minor importance (51, 52). Moreover, velocity information would be crucial to stabilise posture during standing on foam support, where the task difficulty is increased and balance is controlled by many muscles acting at several joints (49, 53–57).

Haptic information is effective in reducing postural sway. The effect of a light fingertip touch is comparable to that obtained by opening the eyes (58–63). It can selectively originate from touch receptors (64) and occurs with contact forces below those necessary to mechanically stabilise the body (65–67). Touch-induced stabilisation occurs both when standing on firm ground and when standing on foam (68, 69) or after a balance perturbation (70, 71). With eyes closed, a light touch of an object next to the body, or a touch of the ground by the cane (66, 72), modifies the control of posture, because finger or cane can be appropriately moved to get the information they are searching for (73). The integration of visual and haptic cues in the control of stance has received much less attention than for the identification of object features (74, 75), but the same operating principles might underpin the effects of either or both inflows. For that matter, reaching and grasping (76) are in fact coordinated with postural adjustments (46, 77).

It is easy to measure sway. The force platform upon which subjects stand captures the path of the wandering centre of foot pressure in a given time period, and its length can be measured along with the surface covered by its journey. The geometric and statistical measures of sway (length of sway path and ellipses containing 95% of the acquired points) show a reasonable reproducibility (78) but bear large inter subject variability (79). Further, although sway path and sway area often co-vary, the correspondence between the former and the latter measure may not be consistent across subjects or patients (27, 80), because the same length of the oscillation skein may not occupy the same surface all the time (81). These measures can also overlap between eyes-open and eyes-closed conditions or between young and elderly (82, 83). In turn, the stabilising effect of vision is indistinguishable from that of touch (62). A different analytical approach might more consistently disclose unique attributes of the visual and haptic stabilising effects (21, 84).

When vision and touch are available, sway can further decrease compared to either information alone (63, 74). On the other hand, removal of peripheral sensation, as by anaesthesia or cooling of the skin of the foot sole, increases body oscillations (40). It might be supposed that integration of multiple inputs, as from the eye, the skin, the proprioceptors, or the graviceptors, can afford excellent body stabilisation (reduction of CoP sway) in accordance with the assumption that “more is better.” This view would implicitly assume the existence of one posture-controlling centre able to integrate the sensory inputs and produce the adequate motor commands, which are evidently optimally designed when the centre receives the best possible amount of information. Body oscillations during quiet stance should then diminish monotonically as a function of the number and competence of the sensory inputs.

The assumption of the present study is that potential differences in the effect of vision or touch on stance control cannot be clearly evinced from the geometric analysis of the standard sway variables such as sway path length or area. Sway metrics more closely connected to the muscle synergies and to the presumably responsible supra-spinal and spinal control modes, expressed by the rambling and trembling behaviour (85, 86), would be more telling. Other methods, like indexing postural dynamics, have been exploited with attention to the multiple time scales of control that subserve standing postures (87, 88), such as the stabilogram-diffusion analysis (89, 90), the wavelet-based spectral analysis (91–93), and the sample entropy (94, 95), which provides measures advising automaticity of postural behaviour.

The purpose of this study has been to increase our knowledge on the role of the sensory control of stance by leveraging the tool of spectral frequency analysis (96–99) rather than through the sole use of geometric sway measures, such as the amplitude of sway area and length of sway path (23). Since oscillations frequencies have a strong relationship to leg and foot muscle activity (100), we hypothesised that the frequencies prevalent under certain conditions may offer a straightforward way of identifying whether the control of stance selects distinct balancing modes under a given sensory condition.

Different laboratories have identified a few frequency windows within the frequency spectrum and have connected these windows to the contribution of vestibular or visual or somatosensory information (99, 101–105). Occasionally, criteria for choosing the width of the frequency windows (106) have been provided. Further, the opinion that proprioception would be disrupted when standing on foam is at variance with the plain consideration that proprioception may be modest and downweighed in a quiet stance (see above), while a massive proprioceptive input must reach the central nervous system during the complex adjustments (often unconsciously produced) carried out when standing on foam (107).

Hence, we addressed the sensory modulation (visual, haptic) of postural behaviour in healthy young people through the use of spectral analysis of the CoP displacement. We have hypothesised that vision and touch stabilise body sway through at least partially different modes of action detectable by the spectral analysis. We first critically examined the use of this tool since there is a wide divergence in the way frequency spectra and frequency windows are defined by different laboratories. We then considered the distribution of the frequency spectrum oscillations when stabilisation was achieved through the use of haptic information (light touch, EC-LT) or vision (EO) or both (EO-LT). In addition, we compared the data obtained by the frequency analysis to those based on geometric sway measures. Finally, we compared the results obtained on the foam to those obtained on a solid base of support (BoS). Consistent modulations of the median frequency of the spectrum and of specific frequency windows thereof emerged, suggesting different neural mechanisms of sway-minimisation strategy for vision and haptic sense.

## Materials and Methods

### Participants

Nineteen healthy young adults (9 men and 10 women) participated in the study. Their average age was 29 ± 4.2 years (mean ± SD), height 172.6 ± 7.2 cm, and weight 68.9 ± 13.5 kg. All subjects were free of neurological and musculoskeletal disorders and either had no sight problems or if so, had their visual acuity corrected during the procedure. All gave written informed consent to participate in the experiments that were performed in accordance with the Declaration of Helsinki and approved by the institutional Ethics Committee (Istituti Clinici Scientifici Maugeri, approval number #2564CE).

### Procedures

Subjects stood barefoot for at least 100 s on a force platform (Kistler 9286BA, Switzerland). The outer profiles of the parallel feet were set at hip width. The head was facing forward. Balance was measured under two Base of Support (BoS) conditions, solid and foam, two visual conditions, eyes open (EO) and eyes closed (EC), and two touch conditions, no-touch and light-touch (LT), resulting in eight experimental conditions. The foot position was marked on a paper sheet placed on top of the platform or of the foam pad (Airex-Balance Pad 50 cm L × 41 cm W × 6 cm H) for consistency across trials.

**Figure 1:**
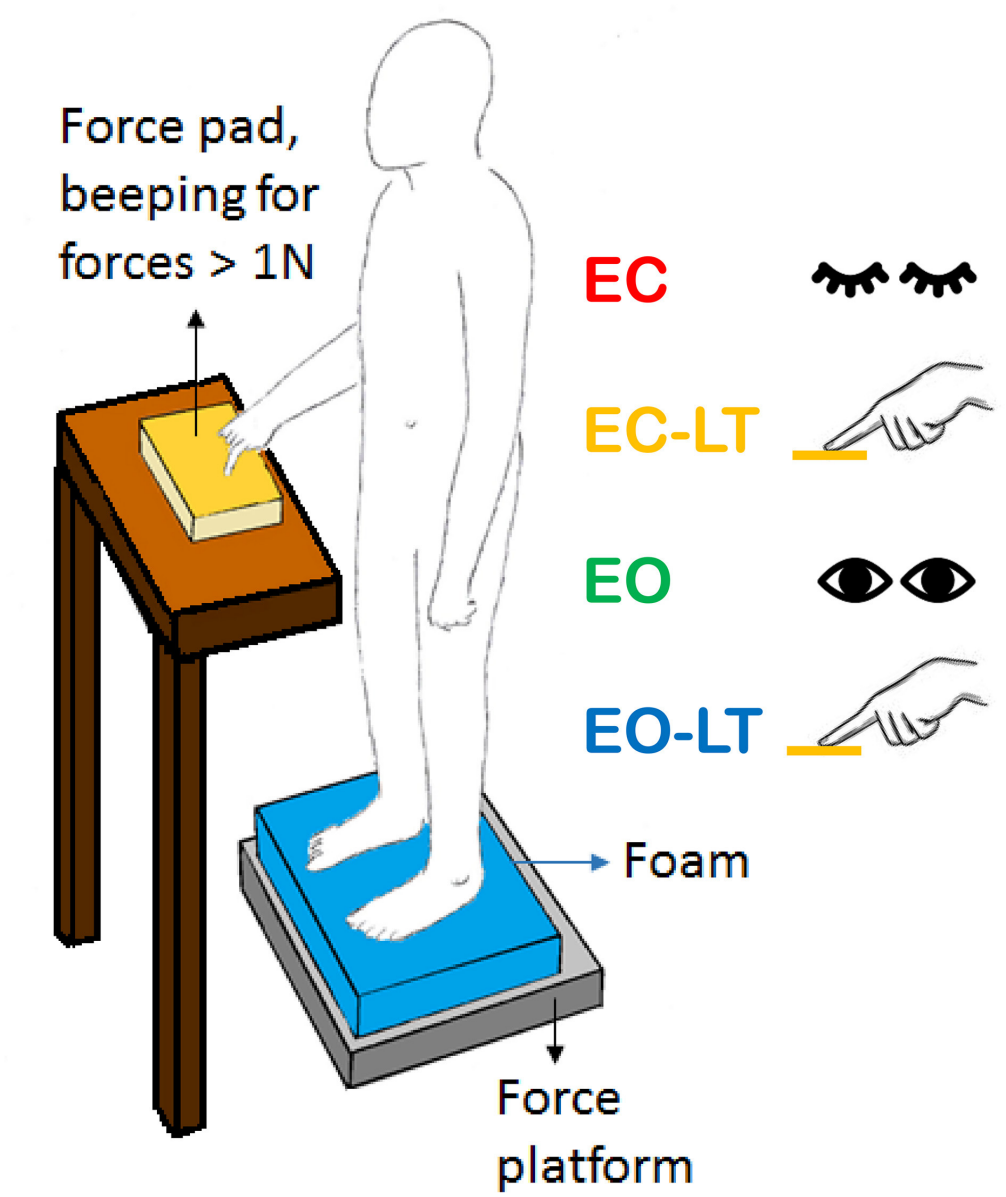
Sketch of the test situation showing a standing subject in front of the force pad (yellow) during the light-touch (LT) trials. The force pad device beeped when the force exceeded 1 N. Subjects rarely reached this force level during the trials. In the sketch, the foam is shown (blue). In the absence of touch, both arms were along the trunk. The same position(s) were assumed with the eyes closed (EC) or open (EO).

Subjects were asked to stand at ease (108), not to stare at a fixed point (109) but to look at the visual scene of the laboratory wall at 6 m distance, featuring the horizontal and vertical profiles of a bookcase. They were asked to avoid the head pitch, roll, and yaw movements, and if possible, ample gaze deviations. In the EC condition, subjects were asked to close their eyes before the start of the acquisition epoch and to keep their eyes closed throughout the trial. In the LT condition, the index finger of the dominant hand was kept on the surface of a haptic device made by a flat horizontal wooden square (10 × 10 cm) fixed on top of a strain gauge (Figure 1). The instruction was to maintain a constant “light touch” on this smooth plane. The output of the strain gauge was recorded by a device that beeped when the vertical force passed the threshold of 1 N. The haptic device was located in front of the subject at about the height of the belly button and distant about 15 cm from it in the sagittal plane. There was no instruction to keep the finger immobile on the force pad and hence, small fluctuations in the hand and finger position were allowed. The finger never slipped off the force pad. The device seldom beeped, mostly in the time period before the acquisition.

The data presented here originate from an investigation that required each volunteer to come to the laboratory eight times on separate days. Each day, the subject completed eight equal-duration (100 s) consecutive standing trials in one of the conditions of interest (EC, EO, EC-LT, and EO-LT). In the following analysis, only the first of the eight trials for each sensory condition has been considered and analysed because an adaptation process proved to take place in the successive trials (manuscript in preparation).

### Data Acquisition and Processing

The last 90s epoch of each 100s stance trial was acquired in order to avoid the accustoming phase occurring immediately after mounting on the platform (with/without foam). This duration of the trials had been selected in order to be the longest possible to avoid exhaustion while at the same time allowing a good resolution of the oscillation frequencies. Critical parameters to obtain a reliable power spectrum were the duration of the acquired epoch (that defines the lowest detectable frequency) and the sampling rate (that defines the highest detectable frequency) (110–112).

All platform data and the data from the haptic device were captured at the sampling frequency of 140 Hz by a PC on which the dedicated software was running (Smart-D, BTS, Italy). All data were moved to another PC for *post-hoc* analysis, and calculations were done using the Excel software and customised LabVIEW programs (National Instrument, USA). The force platform signals of the CoP displacements along both the anteroposterior (AP) and mediolateral (ML) directions were high-pass filtered at 0.01 Hz with a 4th order Butterworth philtre after removing the respective mean values. The length of the sway path was the total length of the wandering CoP during the 90 s epoch, calculated using a software compiled on LabVIEW, and sway area was the surface of the 95% ellipse fitted to the dispersion of the time-series of the AP data plotted against those of the ML recorded in the same epoch (113).

The frequency analysis was performed by applying the fast Fourier transform to the ML and AP CoP time-series data of each trial, subject, sensory, and BoS conditions. This was done by means of the Auto power spectrum VI algorithms of the LabVIEW functions. The frequency resolution, i.e., the sampling frequency (140 Hz) divided by the number of samples acquired by the platform (12,600 samples), was 0.011 Hz. The power spectrum signal was expressed as ${\text{cm}}_{\text{rms}}^{2}$ since the root mean square (rms) of a signal is defined by *u*_*rms*_ = $\sqrt{\frac{1}{T}\int_{0}^{T}{u\left(t\right)}^{2}*t}$. For example, in the case of a sinusoidal waveform like u (t) = A sin (2π/T^*^t), where A is the peak amplitude, T = 1/f, and f is the waveform frequency, the rms of this waveform is u_rms_ = A/√2 = 0.707^*^A. In the case of a sinusoidal peak to peak displacement of 10 cm amplitude, the amplitude of the power spectrum signal would be about 18 ${\text{cm}}_{\text{rms}}^{2}$.

**Figure 2:**
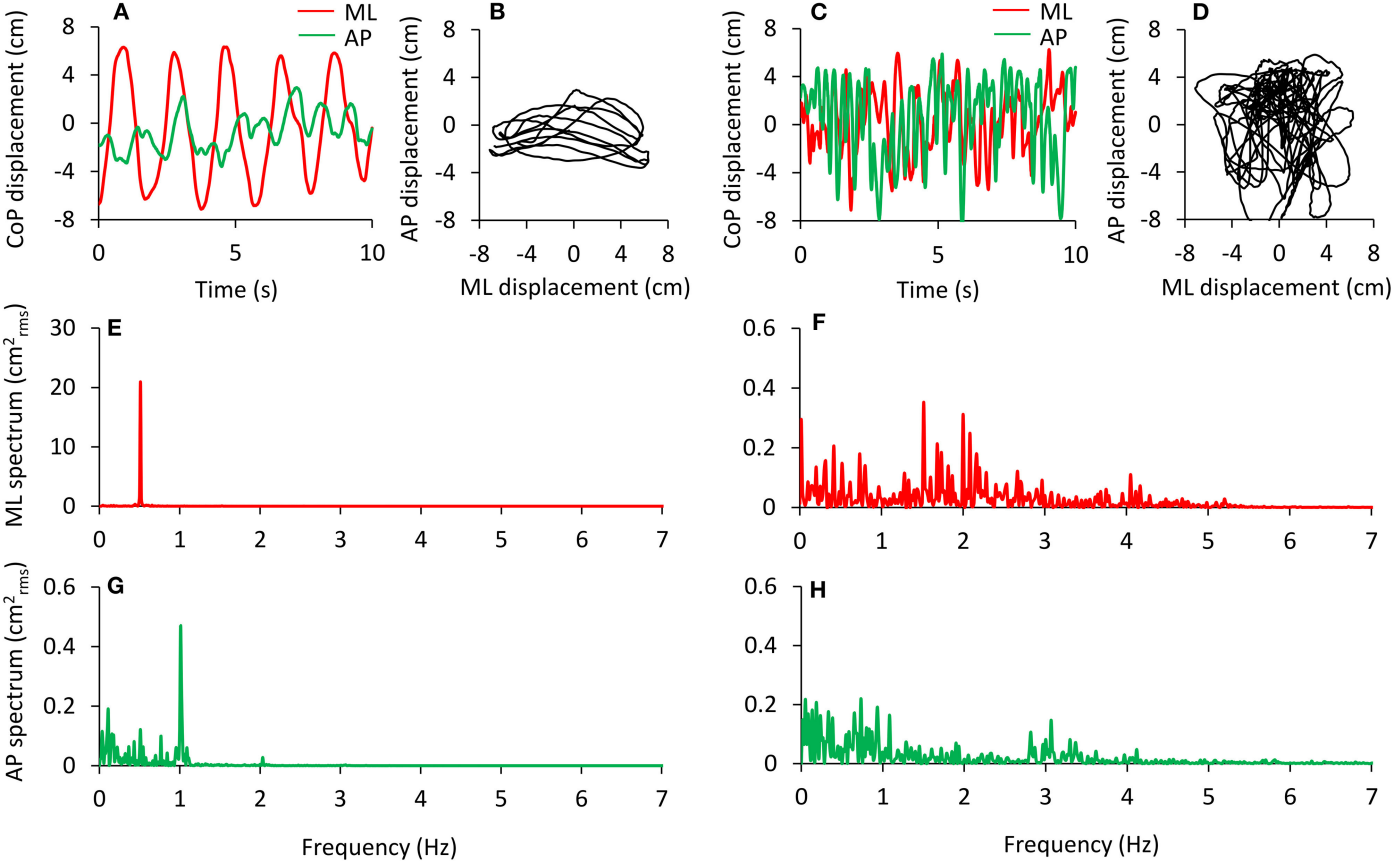
Examples of deliberate sway of the body are performed in order to test the recording and analysis procedures. Voluntary, wide, and rhythmic predominantly mediolateral oscillations were performed in response to 0.5 Hz beeps of a metronome **(A,B)**. The relevant power spectrum profiles in the frontal (ML, with a large peak at 0.5 Hz) and sagittal (AP) planes are reported in **(E,G)**. Note the difference in the ordinates of **(E,G)**. The same subject deliberately performed large body movements at the highest frequency possible **(C,D)**. The relevant power spectrum profiles are shown in **(F,H)**. Now the oscillation frequencies have a wider range and smaller peak amplitudes. Under both circumstances, the deliberate oscillations were performed with the eyes open and lasted 90 s. In **(A–D)**, only 10 s are depicted for easily identifying the CoP oscillations. The traces are dispersed around zero because their mean value has been removed. The power spectra are computed over the 90 s trial.

An example of this analysis is shown in Figure 2. The analysis has been applied to the data of a pilot test made under dynamic EO foam condition consisting of continuous deliberate mediolateral oscillations around 0.5 Hz (left panels) performed by one experimenter following the rhythm of a metronome for a 90 s period. The amplitude of the rhythmic ML displacement was set by the distance between the feet. The computed oscillation frequency in the ML direction (Figure 2A, red) has a peak around 0.5 Hz, and the amplitude of this peak is about 21 ${\text{cm}}_{\text{rms}.}^{2}$ In the AP direction (green), the oscillations are smaller and less regular than in the ML direction with a peak frequency of about 1 Hz (Figure 2G). In the right panels of the Figure, the results of the same analysis are reported and applied to a performance during which the same subject was asked to deliberately shake like a raving lunatic on the foam for 90 s (Figures 2C,D). In this case, the spectral analysis shows oscillations at frequencies >2 Hz, and the amplitude of the spectrum is negligible above 5 Hz (Figures 2F,H).

In the analysis of our experimental trials performed under quiet stance, the frequency range of interest was not predefined. However, we decided to limit the analysis to the part of the frequency spectrum below 2 Hz owing to the negligible amplitude of the power spectrum from 2 Hz onwards. In the EC foam condition, the area under the profile (calculated as the sum of the amplitude of the values of every sample) of the spectrum from 0.01 to 2 Hz corresponded to the 98.8 and 99.0% of the area of the entire spectrum from 0.01 to 70 Hz, for ML and AP, respectively (114).

**Figure 3:**
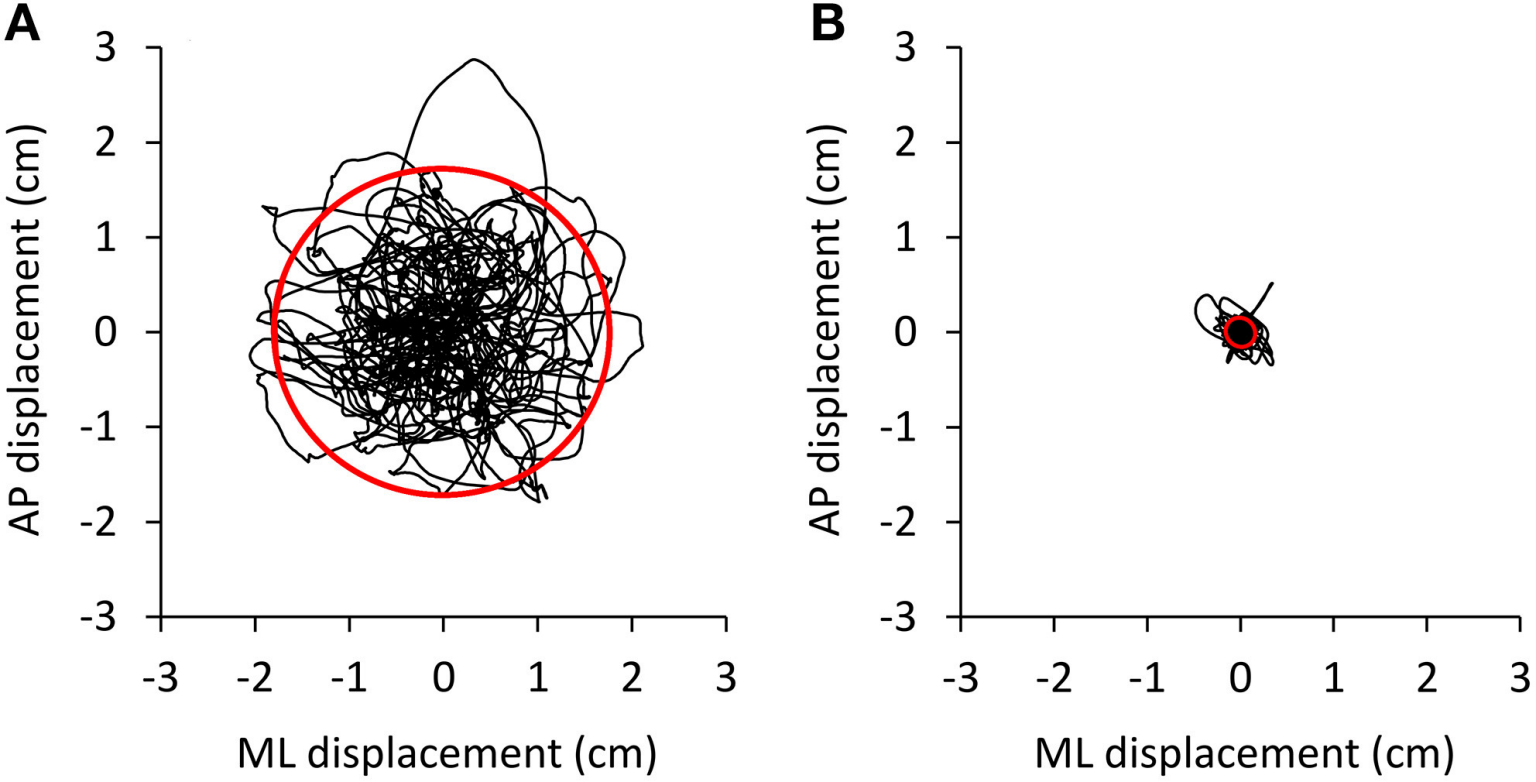
Effect of high-pass filtering. Body oscillations during a 90 s trial were performed with EC on the foam base of support by a representative subject. The oscillations normally ranged about 3–4 cm in both mediolateral (ML) and anteroposterior (AP) directions **(A)**. **(B)** The shrink of the oscillation pattern of the same trial as in A when the AP and ML time series were computed on the 2 Hz high-pass filtered signals.

The amplitude of the body sway (area of the 95% confidence ellipse fitted to the CoP path in the horizontal plane) was hardly affected by oscillation frequencies beyond 2 Hz. Figure 3 shows that the ellipse fitted onto the CoP of the ML and AP traces plotted after high-pass filtering at 2 Hz (Figure 3B) contains a very small percentage (0.7%) of the original unfiltered signal (Figure 3A). The CoP path length diminishes to a much smaller extent (52%).

**Figure 4:**
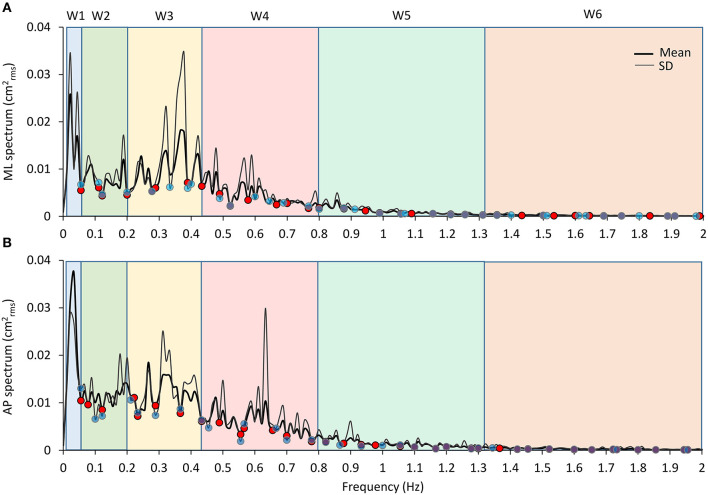
Frequency windows identification. The graphs show the mean profile of the power spectrum (average of the profiles obtained in all the subjects, the thick black line) in the ML and AP directions [**(A,B)**, respectively]. The thin dark grey lines are the corresponding profiles of the SD of the means. A similar pattern for the ML and AP spectra is obvious. The superimposed SD traces largely reproduce the up and downs of the mean spectra and show minimum values close to the relative minimum values of the mean spectra. The blue and red dots correspond to the local minima of the mean and SD traces, respectively, computed in the consecutive 0.05 Hz windows. The pale-coloured rectangles indicate the frequency windows used in the analysis.

Median frequency and mean level of the spectrum were calculated for each sensory and BoS condition between 0.01 and 2 Hz for the AP and the ML CoP displacements. Median frequency (at which the power spectrum is divided into two parts of equal area) was calculated by means of Matlab software. Then, specific frequency windows (Ws) were identified for further analysis of the effects of the manipulation of conditions on the power spectrum. The Ws identification was made based on the “default” condition (the EC foam trial), which featured the maximum overall amplitude of the entire power spectrum profile compared to all other conditions tested. Then, the boundaries of the Ws were selected based on the profile of the mean power spectrum obtained by averaging the profiles of the EC foam trial of all the subjects (Figure 4). In detail, the Ws have been operationally identified by the superimposition of the mean EC power spectra separately for the frontal (ML) and the sagittal (AP) planes. The local *minima* were identified in successive epochs of 0.05 Hz of the traces. The same procedure was repeated for the local *minima* of the profile of the SD trace of the mean power spectrum as a companion criterion. It turned out that the local *minima* in the successive epochs of the mean power spectrum trace and of its SD trace almost coincided in most cases. These points would correspond to oscillation frequencies poorly represented in our population. They were arbitrarily considered critical discriminating points for the identification of the boundaries of the Ws. Further, in order to simplify the interpretation, some of the *minima* were disregarded and a few adjacent Ws merged. Hence, the analysis was restricted to six windows only.

Moreover, based on the visual comparison of the profiles of the mean power spectra obtained for the ML and AP directions of CoP oscillations and of their SD, which were similar and almost superimposable across the entire frequency range, we decided to utilise the same Ws identified for the ML direction for the AP direction. This procedure allowed to identify the following frequency Ws, which were equal for both the ML and the AP directions: W1 (the lowest frequency), from 0.01 to 0.055 Hz; W2, 0.055 to 0.2 Hz; W3, 0.2 to 0.44 Hz; W4, 0.44 to 0.8 Hz; W5, 0.8 to 1.31 Hz; and W6, 1.31 to 2 Hz. For each W, the mean level of the spectrum was calculated and compared across the sensory and BoS conditions.

### Data Treatment and Statistics

The mean power spectra profiles (mean + SD) obtained for the CoP oscillations were point-by-point compared by Student's *t*-test according to a procedure used in this laboratory (115). The oscillation frequencies at which the Student's *t*-test value bypassed the probability of 0.05 (two-tailed pairwise test) were taken as the frequencies at which ML and AP oscillations became different. This procedure was used to compare the ML and AP power spectra in the EC condition and the spectra under both EC-LT and EO conditions on foam in order to investigate the differences in the contribution to the stabilisation process of touch and vision.

The data pertaining to the mean profile of the AP power spectrum were plotted against those of the mean profile of the ML power spectrum for the EC foam condition. This relationship was studied by a linear regression model and the coefficient of determination (*R*^2^) was calculated. Also, the relationships between the mean level of the ML and AP spectrum in each frequency window and the CoP path length and sway area were studied by a linear regression model and the *R*^2^ was calculated.

Assumptions for parametric statistics were met for all variables of interest as assessed by the Kolmogorov-Smirnov and Levene's test. The following analyses were performed separately for the two BoS conditions (solid and foam). A 2 (ML and AP directions) × 4 (vision and touch conditions) repeated measure (rm) ANOVA was used to compare the median frequency and the mean level of the spectrum between 0.01 and 2 Hz. A 2 (ML and AP directions) × 4 (vision and touch conditions) × 6 (frequency windows) rm ANOVA was used to compare the mean level of the spectrum calculated in each frequency window. In order to highlight the difference between sensory conditions in each window, a 2 (ML and AP directions) × 4 (vision and touch conditions) rm ANOVA was applied to the mean level, separately for each window. The effects of the different sensory conditions on path length and sway area of the CoP were compared by a 1-way rm ANOVA. A two-tailed paired *t*-test was used to compare the force exerted by the subjects on the touch pad between EC-LT and EO-LT conditions.

The main effects of BoS (foam and solid) on the median frequency and on the mean level of the spectrum between 0.01 Hz and 2 Hz were compared by a 2 (BoS) × 2 (ML and AP directions) × 4 (vision and touch conditions) rm ANOVA. A 2 (BoS) × 4 (vision and touch conditions) rm ANOVA was used to test the effect of the two BoS on CoP path length and sway area. A 2 (BoS) × 2 (EC-LT and EO-LT conditions) rm ANOVA was used to compare the effect of the base of support on the force exerted by the subjects on the touch pad. The *post-hoc* was the Fisher's LSD test. The significance level was set at 0.05. The value of $\eta_{\text{p}}^{2}$ was reported as well. Where the differences were significant, the Cohen's d effect sizes highlighted the strength of the difference. Statistical tests were performed using Statistica (Statsoft, USA).

## Results

The findings are itemised for the sake of clarity. The CoP data collected in the foam condition are presented first, followed by those recorded in the solid BoS condition. Within each branch of the investigation, the power spectrum data in the different experimental conditions are presented first, followed by the geometric data of path length and sway area and by the comparisons of frequency and geometric data. In both cases, the data regarding the ML precede those of the AP oscillations. The comparisons between foam and solid BoS conditions are reported at the end of the section.

### Foam Base of Support

#### Power Spectrum

##### ML and AP Oscillations (EC) Have Similar Profiles

The analysed range of the power spectrum reached from 0.01 to 2 Hz. The area under the curve of this range corresponded to more than 98% of that of the entire spectrum (along both the ML and the AP directions). In particular, no frequency peak however small was obvious in the profile of the power spectrum beyond 2 Hz.

**Figure 5:**
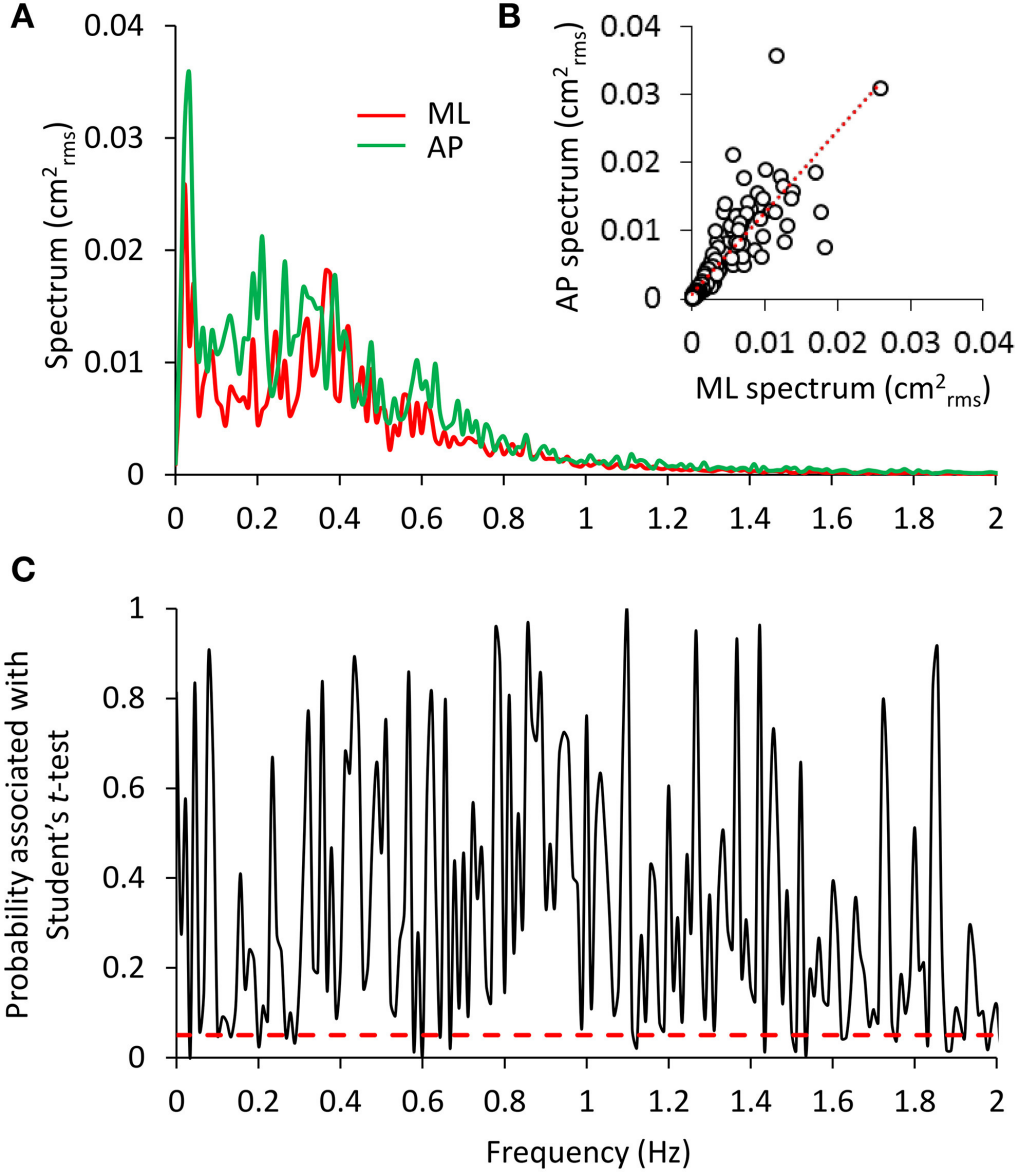
Comparison between the ML and AP spectra. In **(A)**, the superimposed power spectra in the ML and AP directions (EC) appear similar, except for a slightly higher amplitude of the AP (green) compared to the ML spectrum (red). The regression line fitted to the data points of AP vs. ML **(B)** has a slope slightly higher than 1 and the intercept close to origin of the axes (y = 1.15 × + 0.0008, *R*^2^ = 0.72, *p* < 0.001). The plot in **(C)** shows the probability values associated with the *t*-test of the point-to-point differences between the two spectra. The red dashed line indicates the significance of the differences at *p* < 0.05. Consistent but limited differences between the two spectra were observed at low frequencies.

Figure 5 shows the mean power spectra superimposed for the ML and AP oscillations (Figure 5A) in the EC condition and the result of applying the *t*-test to each of the frequency values (Figure 5C). The profiles were similar. However, differences between the two spectra were detected by the Student's *t*-test between 0.1 and 0.3 Hz and in scattered positions for higher frequencies. In inset Figure 5B, the data pertaining to the mean profile of the AP power spectrum were plotted against those of the mean ML power spectrum for the EC condition. Each sampled frequency point is considered (*n* = 180 data points corresponding to the frequency units). Clearly, there is a good regression line, indicating that when the value of the power spectrum profile at a certain sampled frequency was low in ML, the corresponding AP value was also low and vice versa.

##### Sensory Conditions

**Figure 6:**
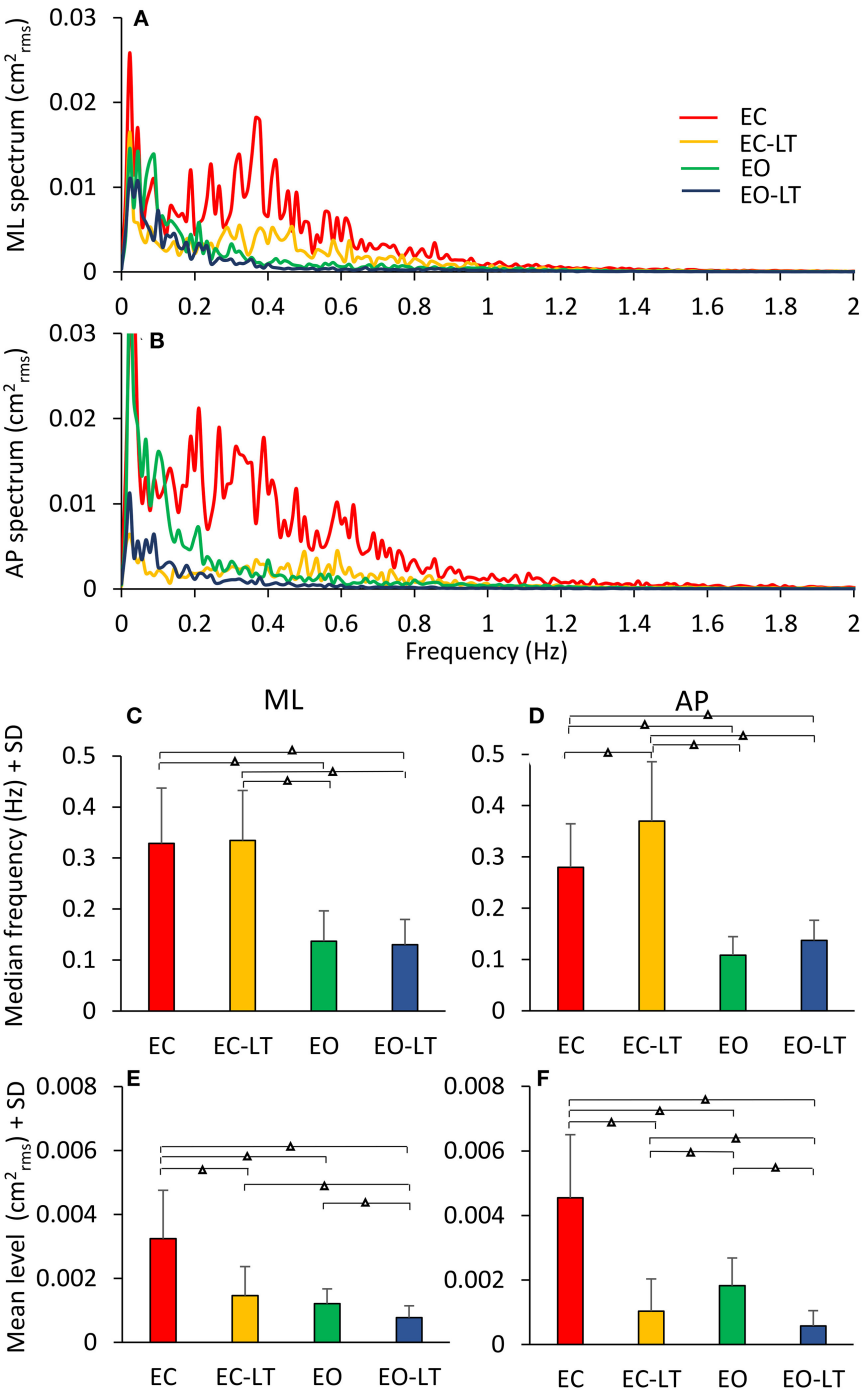
Power spectra under different sensory conditions. This contrasts the spectra during the trials performed on foam without vision (EC, red), with vision (EO, green), and with the addition of touch (EC-LT, yellow; EO-LT, blue). Obviously, the level of the spectrum for all the “stabilised” conditions is smaller than with EC **(A,B)**. The median frequency of the spectrum **(C,D)** is definitely smaller with vision (EO and EO-LT) than without vision (EC and EC-LT), both in the frontal and sagittal plane. Conversely, the mean level **(E,F)** is small in the three “stabilised” conditions. In this case, the reduction in the mean level also applies to EC-LT. Triangles indicate significant differences (^**Δ**^*p* < 0.001).

The mean profiles of the power spectra in the four tested conditions (EC, EC-LT, EO, EO-LT) when standing on foam are shown in Figures 6A,B. For each condition and each subject, the median frequency and the mean level of the spectrum was calculated (Figures 6C–F). All conditions included, the median frequency was not different between ML and AP [*F*_(1, 18)_ = 0.86, *p* = 0.36]. However, there was a significant difference in the median frequency between sensory conditions [*F*_(3, 54)_ = 64.5, *p* < 0.001, *d* = 3.76, $\eta_{\text{p}}^{2}$ = 0.78] and a significant interaction between ML and AP directions and sensory conditions [*F*_(3, 54)_ = 3.16, *p* = 0.03, *d* = 0.84, $\eta_{\text{p}}^{2}$ = 0.15]. For both ML and AP, the median frequency was higher with EC (EC and EC-LT) than with EO (EO and EO-LT) (*post-hoc, p* < 0.05 for all comparisons). In the ML direction, there was no difference in the median frequency between EC and EC-LT (*p* = 0.76) and between EO and EO-LT (*p* = 0.73). In the AP direction, instead, the median frequency was higher with EC-LT than with EC (*p* < 0.001), but there was no significant difference between EO and EO-LT (*p* = 0.14). All conditions included, there was a significant difference in the mean level of the spectrum between ML and AP directions [*F*_(1, 18)_ = 5.53, *p* = 0.03, *d* = 1.1, $\eta_{\text{p}}^{2}$ = 0.23], a significant difference between sensory conditions [*F*_(3, 54)_ = 88.35, *p* < 0.001, *d* = 4.43, $\eta_{\text{p}}^{2}$ = 0.83] and a significant interaction between ML and AP directions and sensory conditions [*F*_(3, 54)_ = 27.11, *p* < 0.001, *d* = 2.45, $\eta_{\text{p}}^{2}$ = 0.6]. In the ML direction, the mean level of the spectrum under EC condition was the highest (*post-hoc, p* < 0.001 for all comparisons) and became the smallest in the EO-LT condition (*p* < 0.01 for all comparisons). In the EC-LT condition, the mean level was not different to the mean level under EO (*p* = 0.1). Also, in the AP direction, the mean level of the spectrum under EC condition was the highest (*post-hoc, p* < 0.001 for all comparisons) and became the smallest in the EO-LT condition (*p* < 0.01 for all comparisons). Moreover, in the EC-LT condition, the mean level of the AP spectrum was smaller than in the EO condition (*p* < 0.001).

**Figure 7:**
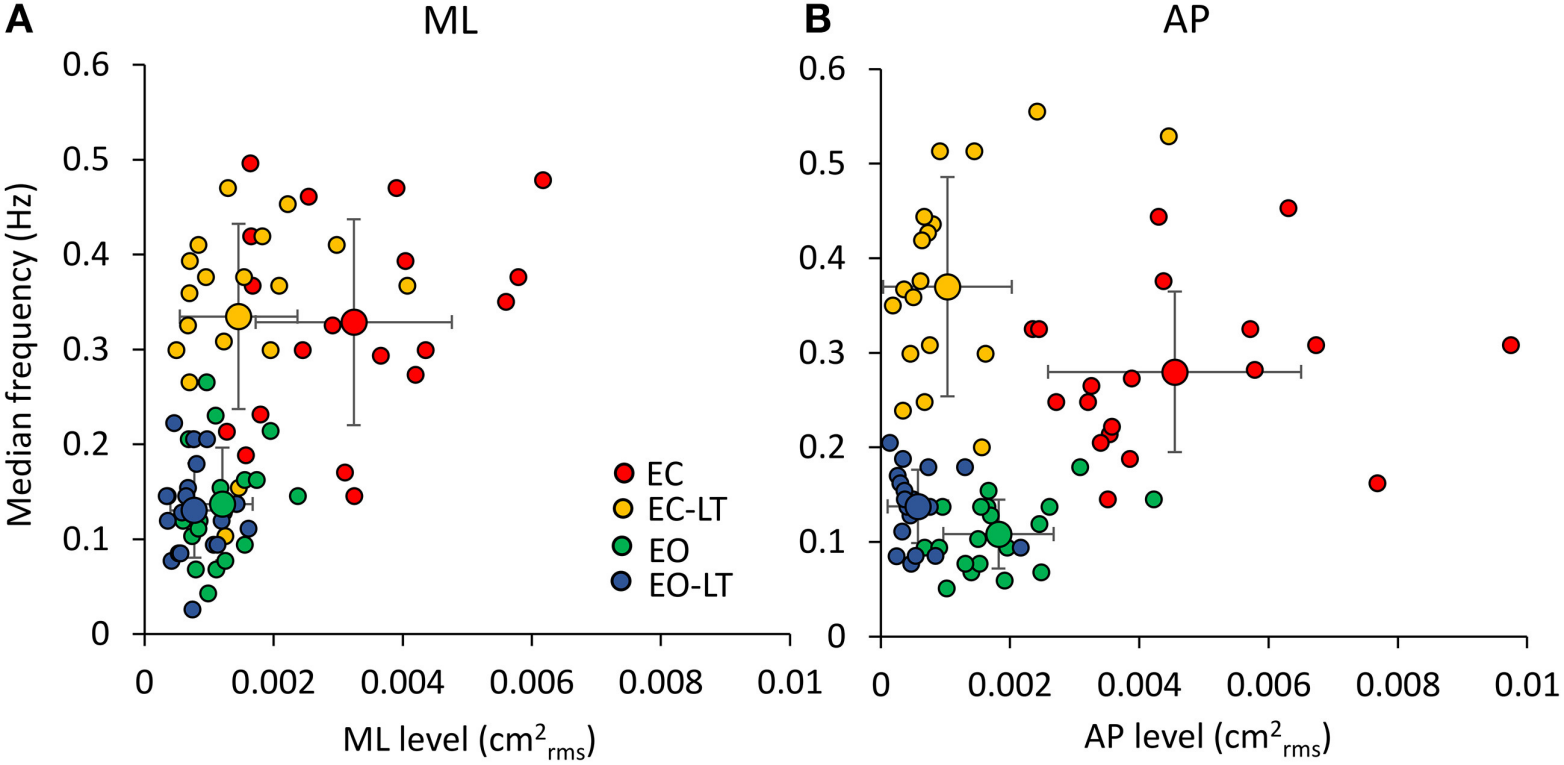
Relationship between median frequency and mean level of the spectrum. There is a weak relationship between the value of the median frequency and the mean value of the entire spectra in each subject (*n* = 19), in both the frontal **(A)** and the sagittal planes **(B)**. The relatively large variability across subjects is underscored by the plots. However, when the mean values of each of the coloured clusters are considered (the large circles with their SDs), it appears that vision (EO, green) and vision and touch (EO-LT, blue) feature small oscillation frequencies with small oscillation amplitudes. Without vision, however, touch clearly reduces the mean level (EC-LT, yellow) compared to no-vision (EC, red), while the oscillation frequencies are hardly changed, particularly along the ML direction.

In Figure 7, the median frequency of the spectrum is plotted against its mean level for ML and AP directions in the four sensory conditions for each subject. A large variability across subjects is obvious in the plots. However, it is clear that the red and yellow circles (no vision, EC and EC-LT, respectively), have high median-frequency values, while the green and blue circles (vision, EO and EO-LT, respectively) mostly include low frequencies values. Conversely, touch (EC-LT and EO-LT, the clusters of the yellow and blue symbols, respectively), produced the largest reduction in the mean level, regardless of the visual condition. This consideration applies to both ML and AP directions. In the plots, the large circles correspond to the mean values of the clusters. As expected, the conditions EO and EO-LT feature small values for both variables. The median frequencies with touch were just larger (even not significantly so for the ML direction) than under the corresponding EC and EO conditions (without touch) for both the ML and the AP directions.

##### Distinct Effects Are Elicited by the Sensory Conditions in the Different Frequency Windows

**Figure 8:**
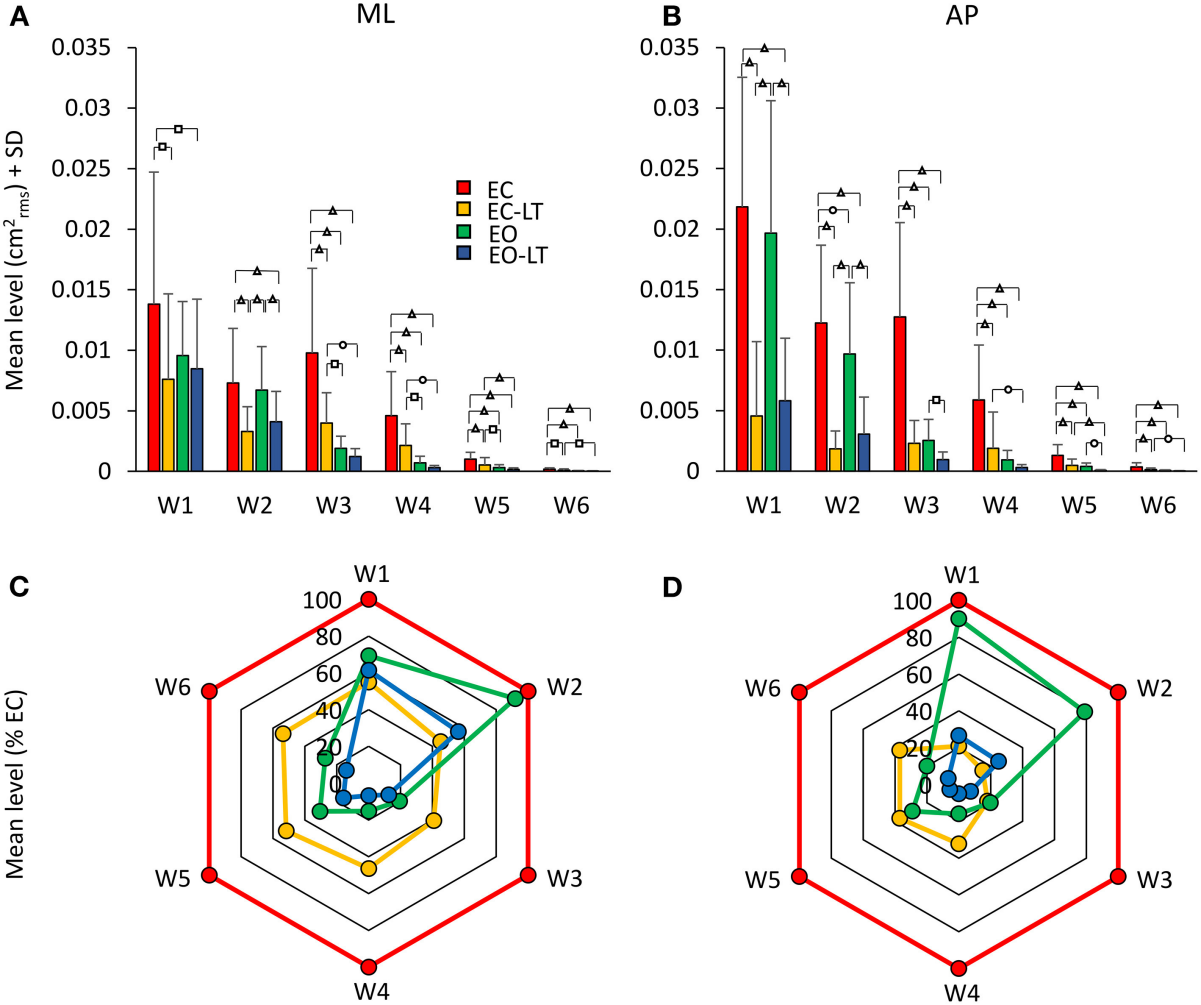
Mean levels of the spectrum in the frequency windows (Ws) standing on foam. The mean level of the spectrum is irregularly distributed across the distinct Ws **(A,B)**. The EC condition (red bars) features a large mean level compared to the other sensory conditions in all the Ws, regardless of the ML or AP direction and of the progressive decrease in amplitude at the higher frequencies. The “stabilised” conditions show a non-uniform pattern depending on Ws and sensory conditions. EC-LT reduces the mean level in all the Ws in ML and more so in AP. Whereas, in ML and AP, EO (green) shows large mean levels at the low frequencies. Overall, the mean level at the highest frequencies is broadly reduced in all “stabilised” conditions. Distinct symbols indicate significant differences (^□^*p* < 0.05; **°***p* < 0.01; ^**Δ**^*p* < 0.001). A compact compendium of the percent reduction compared to EC (red outermost trace) in the mean level of the distinct frequency Ws under the EC-LT, EO, EO-LT conditions is given by the radar plots in **(C)** (ML) and **(D)** (AP).

Figure 8 shows the mean level of the spectrum calculated for the ML (Figure 8A) and AP directions (Figure 8B) across all the subjects in each of the identified frequency windows. In the bottom panels (Figures 8C,D), the percent changes with respect to the EC condition are reported for the ‘stabilised’ conditions. There was a difference between the mean level of the spectra of the ML and AP directions, all conditions included [*F*_(1, 18)_ = 13.1, *p* < 0.01, *d* = 1.7, $\eta_{\text{p}}^{2}$ = 0.42], a difference between sensory conditions [*F*_(3, 54)_ = 96.6, *p* < 0.001, *d* = 4.62, $\eta_{\text{p}}^{2}$ = 0.84] and between frequency windows [*F*_(5, 90)_ = 115.6, *p* < 0.001, *d* = 5.06, $\eta_{\text{p}}^{2}$ = 0.86]. There was a significant interaction between ML and AP directions and sensory conditions [*F*_(3, 54)_ = 22.2, *p* < 0.001, *d* = 2.23, $\eta_{\text{p}}^{2}$ = 0.55], ML and AP directions and frequency windows [*F*_(5, 90)_ = 3.4, *p* < 0.01, *d* = 0.87, $\eta_{\text{p}}^{2}$ = 0.16], between sensory conditions and frequency windows [*F*_(15, 270)_ = 15.4, *p* < 0.001, *d* = 1.83, $\eta_{\text{p}}^{2}$ = 0.45], and between ML and AP directions, sensory conditions and frequency windows [*F*_(15, 270)_ = 5.26, *p* < 0.001, *d* = 1.08, $\eta_{\text{p}}^{2}$ = 0.23].

Figures 8C,D give an easy view of the similarities and differences in the mean levels of the distinct frequency windows in the three “stabilised” conditions. In W1 and W2, vision (EO, green) had a small effect in ML and AP compared to EC. For all remaining frequency windows, vision reduced the mean level to a large extent in AP and ML. Touch without vision (EC-LT, yellow) moderately reduced the mean level in all frequency windows in ML, more so in AP. Touch and vision (EO-LT, blue) reduced the mean level of the entire spectrum in ML, particularly for the W3–W6, more so in AP.

*ML Direction*. In detail, in W1, the mean levels in the EC and EO conditions were not much different (*post-hoc, p* = 0.09). The mean level with EC was >2 conditions with touch (EC-LT and EO-LT, *p* < 0.05 for both comparisons). When touch was added (EC-LT condition), the mean level of the spectrum was not different from the mean level of the EO and of the EO-LT conditions (*p* > 0.4 for both comparisons). When touch was added to vision (EO-LT), there was no difference compared to the EO condition (*p* = 0.67). In W2, touch diminished the mean level of the spectrum with respect to the corresponding visual condition without touch (*post-hoc*, EC-LT vs. EC: *p* < 0.001; EO-LT vs. EO: *p* < 0.05). Touch under the EC condition diminished the mean level with respect to EO (*p* < 0.01). EC and EO were not different (*p* = 0.6). In W3, EC had the largest spectrum than the other sensory conditions (*p* < 0.001 for all comparisons). When touch was added (EC-LT), the spectrum diminished to less than half of EC (*p* < 0.001) but remained greater than EO (*p* < 0.05) and EO-LT (*p* < 0.01). When touch was added to vision, there was no difference in the amplitude of the spectrum compared to EO (*p* = 0.33). In W4, the EC had the largest spectrum compared to the other sensory conditions (*p* < 0.001 for all comparisons). With EC-LT, the mean level was greater than with EO and EO-LT (*p* < 0.05 for both comparisons). There was no difference between EO and EO-LT (*p* = 0.51). In W5, EC was greater than EO (*p* < 0.05). When touch was added (EC-LT), the mean level was smaller with respect to EC (*p* < 0.001). There was no difference between EO and EO-LT (*p* = 0.14). In W6, the mean level in the EC condition was the greatest (*p* < 0.05 for all comparisons).

*AP Direction*. In W1, touch with respect to no-touch diminished the mean level of the spectrum both with EC and EO (*post-hoc, p* < 0.001 for all comparisons). There was no difference between EC and EO either with (EC-LT vs. EO-LT, *p* = 0.62) or without touch (EC vs. EO, *p* = 0.39). The mean level in W2 behaved similarly to W1. Again, touch diminished the mean level of the spectrum with respect to the corresponding visual condition without touch (EC-LT vs. EC, *p* < 0.001; EO-LT vs. EO: *p* < 0.01). The mean level without vision (EC) was reduced by a touch more than by vision (*p* < 0.001). There was a significant difference in this window between EC and EO (*p* < 0.01), but there was no difference between EC-LT and EO-LT (*p* = 0.18). The pattern of the spectrum in the four sensory conditions was broadly reproduced in W3, W4, W5, and W6. EC had the largest mean level than the other sensory conditions (*p* < 0.001 for all comparisons within each window). When touch was added (EC-LT), the spectrum became similar to EO (*p* > 0.08 in each window). There was a difference between EO and EO-LT in W3 and W5 (*p* < 0.05 for both windows) but not in W4 and W6 (*p* > 0.14 for both windows).

##### Touch and Vision

**Figure 9:**
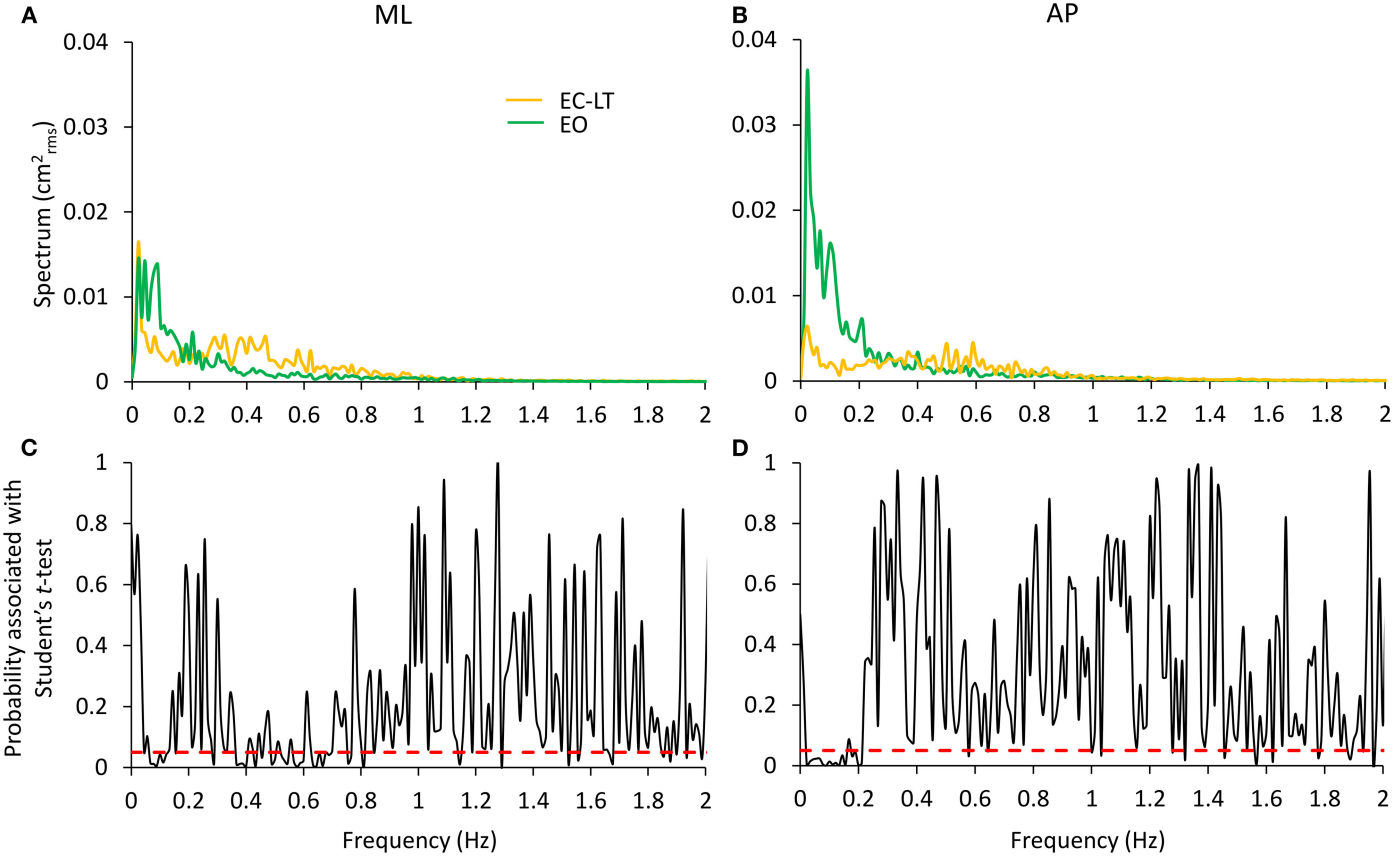
Comparison of the effects of the “stabilising” touch and vision conditions. EC-LT (yellow) and EO (green) are compared in **(A,B)** for the ML and AP directions, respectively. Touch compared to vision remarkably decreases the spectrum in the very low frequency range (~0.01–0.2 Hz) but increases its level in the range 0.3–0.8 Hz. Graphs **(C,D)** (reporting the *p*-values of the point-to-point differences between the mean values of the entire spectra) show that the mean levels are significantly different in these ranges. The differences are less strong in the higher frequency range for the AP direction. The red dashed lines indicate the level at which the differences are significant at *p* < 0.05.

The potentially different processes subserving the “stabilising” effects of touch without vision (EC-LT) and of vision without touch (EO) have been the object of additional analysis. In Figure 9, the spectrum of the EC-LT condition is superimposed to that of the EO condition for the ML (Figure 9A) and AP (Figure 9B) directions, and the result of applying the point-by-point *t*-test analysis (Figures 9C,D) is shown. For the ML direction, touch (EC-LT) significantly decreased the amplitude of the spectrum between 0.07 and 0.15 Hz (approximately corresponding to the frequency window W2) with respect to the EO (no-touch), while the amplitude of the spectrum was greater for EC-LT with respect to EO between 0.3 and 0.8 Hz (approximately corresponding to W4). For the AP direction, the amplitude of the spectrum was smaller with EC-LT than EO only at low frequency, while there was an increased amplitude for frequencies in W4 (EC-LT > EO). This analysis confirms the emergence of a relatively large high-frequency component of the spectrum, for both ML and AP, selective for touch. However, this component did not raise the spectrum up to its EC values and disappeared when vision was available (EO-LT, see Figures 5, 7).

#### Geometric Sway Measures

**Figure 10:**
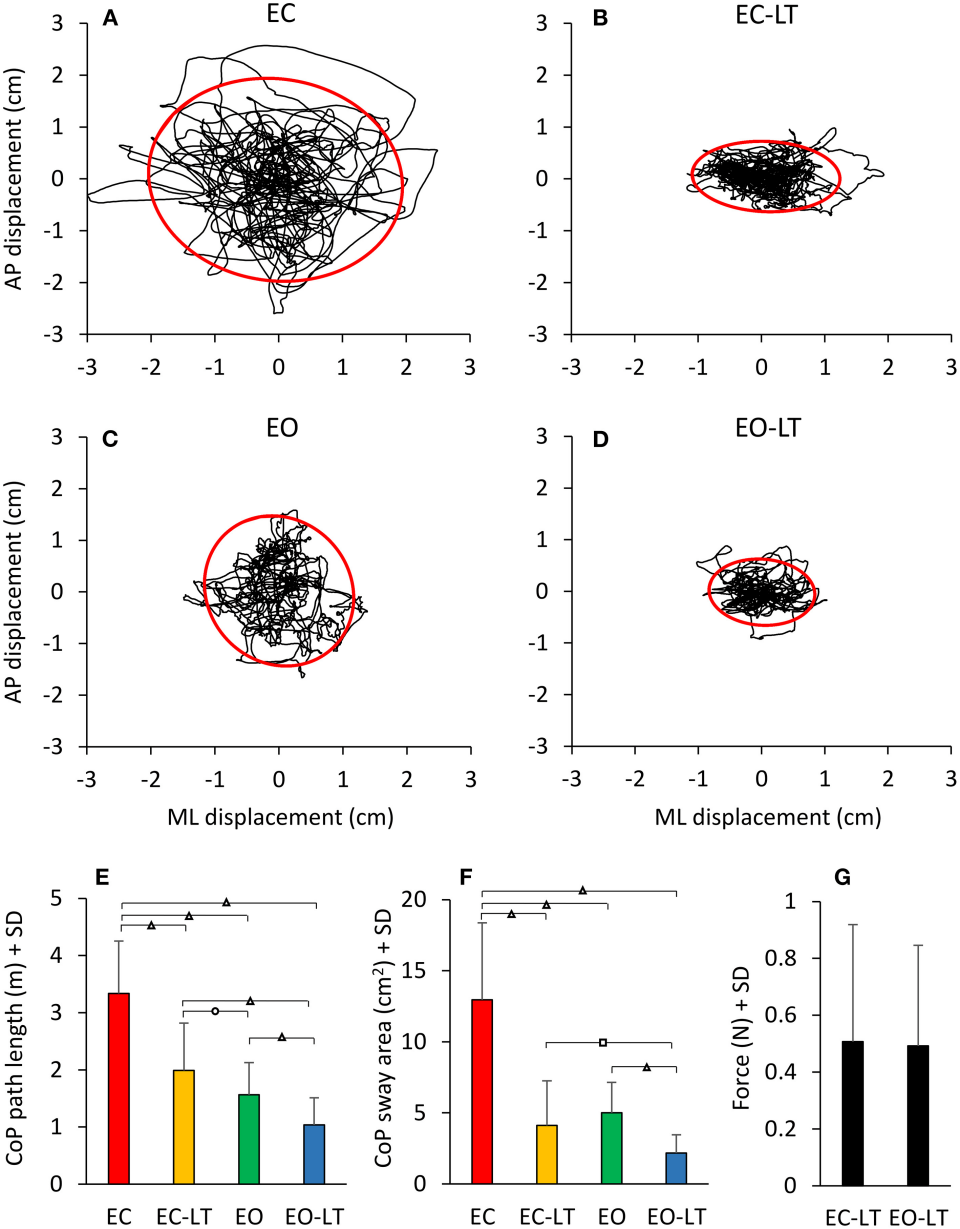
Geometric sway measures. The four top panels show representative diagrams of the CoP sway trajectory (black line) together with the 95% ellipse profile (red) in one subject in the four sensory conditions tested on foam **(A–D)**. The diagrams broadly match the mean values of path length **(E)** and sway area **(F)** of the entire cohort of subjects. There is a discordance in the two metrics (length and area), whereby the path length diminishes with EC-LT and EO-LT, but the sway area decreases proportionally more. **(G)** The fingertip forces applied to the force pad during the touch conditions (both EC-LT and EO-LT) were similar. Distinct symbols indicate significant differences (^□^*p* < 0.05; **°***p* < 0.01; ^**Δ**^*p* < 0.001).

The statokinesigrams of a representative subject under the four different conditions while standing on the foam BoS are shown in Figures 10A–D. Sway area diminished with touch and with vision with respect to EC. The bottom panels show the mean CoP path length (Figure 10E) and the mean sway area (Figure 10F) calculated across all subjects. The CoP path length was different between conditions [*F*_(3, 54)_ = 18.4, *p* < 0.001, *d* = 4.76, $\eta_{\text{p}}^{2}$ = 0.85] and there was a difference between each condition (EC > EC-LT > EO > EO-LT, *post-hoc, p* < 0.01, for all paired comparisons). When vision and touch were combined (EO-LT), the CoP path length became the smallest (*p* < 0.001 for all paired comparisons). Of note, the force applied by the fingertip to the force pad (Figure 10G) was virtually the same regardless of the vision condition (EC-LT and EO-LT, paired *t*-test, *p* = 0.63).

Similar but not identical results were obtained considering the sway area. There was a significant difference between conditions [*F*_(3, 54)_ = 83.4, *p* < 0.001, *d* = 4.3, $\eta_{\text{p}}^{2}$ = 0.82]. With EC, the sway area was the largest with respect to the other sensory conditions (*post-hoc, p* < 0.0001 for all comparisons) and became the smallest with EO-LT (*p* < 0.0001 for all comparisons). When touch was added to EC, the sway area become not different from EO (EC-LT vs. EO, *p* = 0.23) but remained greater than with EO-LT (*p* < 0.001).

**Figure 11:**
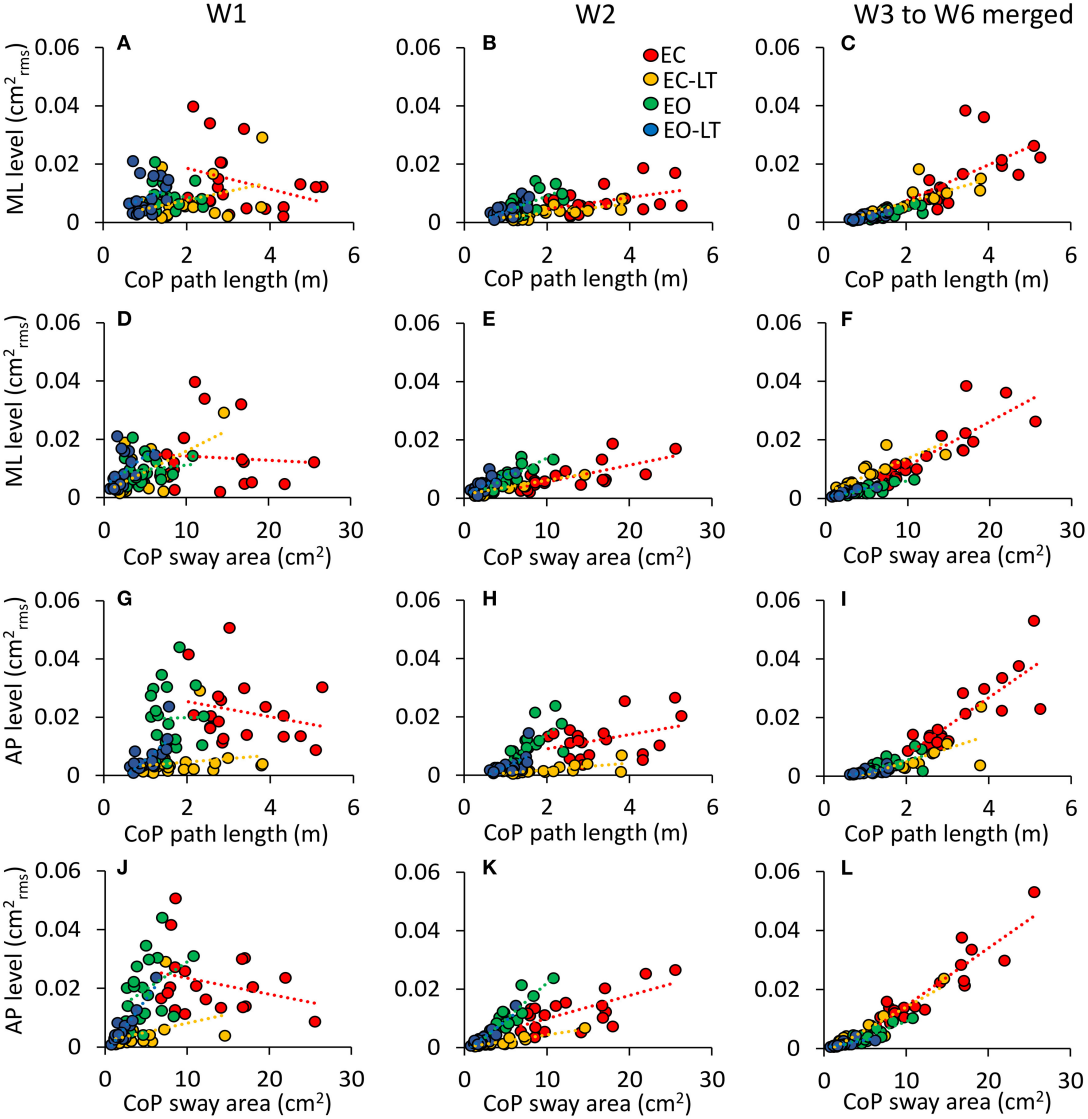
Not all the frequency windows match the geometric sway measures. The contribution of the ML **(A–F)** and AP **(G–L)** sway frequencies to the geometric sway measures is limited to high frequency windows (EC, red; EC-LT, yellow; EO, green; EO-LT, blue). The columns refer to W1 **(A,D,G,J)**, W2 **(B,E,H,K)**, and from W3–W6 **(C,F,I,L)**. Each symbol refers to one subject. **(A–C,G–I)** The spectrum amplitudes vs. the CoP path length, and **(D–F,J–L)** the spectrum amplitudes vs. the sway area. For both sway area and path length, the slope of the regression lines increases with frequency under all sensory conditions but only for W2 onwards.

The relationship between the mean level of the spectrum in the ML (Figures 11A–F) and AP directions (Figures 11G–L) vs. the CoP path length and sway area in the different frequency windows are shown in Figure 11. W1 and W2 are shown in the first two columns. In the panels of the right column, the frequency windows from W3 to W6 (containing the highest frequencies) and their levels are merged. There was no relationship between the mean level of the ML or AP spectrum and the CoP path length or sway area in the first frequency window (W1). The relationship improved from the W2 to the higher frequency windows, especially for the CoP sway area (*p* < 0.001 for all regression lines) for both ML and AP directions. The equation of the regression lines fitted to the data is reported in Table 1 for the ML and AP directions.

**Table 1:** Relationship between level of mediolateral (ML, left part) and anteroposterior (AP, right part) spectrum and CoP path length and sway area.

**FW**	**Condition**	**ML spectrum vs. CoP path length**			**ML spectrum vs. sway area**			**AP spectrum vs. CoP path length**			**AP spectrum vs. sway area**		
**Entire spectrum (from 0.01 to 2 Hz)**		**Equation**	***R^**2**^***	***p*-value**	**Equation**	***R^**2**^***	***p*-value**	**Equation**	***R^**2**^***	***p*-value**	**Equation**	***R^**2**^***	***p*-value**
	EC	y = −0.001x−0.00006	0.41	<0.01	y = 0.0003x−0.0001	0.85	<0.001	y = 0.0016x−0.0009	0.7	<0.001	y = 0.0003x + 0.0003	0.8	<0.001
	EC LT	y = 0.0009x−0.0003	0.68	<0.001	y = 0.0003x + 0.0003	0.89	<0.001	y = 0.0009x−0.0007	0.58	<0.001	y = 0.0003x−0.0002	0.94	<0.001
	EO	y = 0.0009x−0.0002	0.54	<0.001	y = 0.0002x + 0.0001	0.94	<0.001	y = 0.0013x−0.0003	0.4	<0.01	y = 0.0004x−0.0002	0.96	<0.001
	EO LT	y = 0.0009x−0.0002	0.56	<0.01	y = 0.0003x + 0.0002	0.74	<0.001	y = 0.0012x−0.0007	0.57	<0.001	y = 0.0004x−0.0002	0.9	<0.001
FW 1	EC	y = −0.0036x + 0.026	0.1	0.2	y = −0.0001x + 0.016	0.005	0.77	y = −0.0026x + 0.03	0.06	0.32	y = −0.0005x + 0.03	0.08	0.25
	EC LT	y = 0.003x + 0.0016	0.13	0.13	y = 0.0014x + 0.002	0.4	<0.01	y = 0.0012x + 0.002	0.03	0.5	y = 0.0006x + 0.002	0.1	0.19
	EO	y = −0.002x + 0.013	0.035	0.44	y = 0.0003x + 0.008	0.02	0.54	y = 0.0005x + 0.02	0.0003	0.95	y = 0.002x + 0.01	0.13	0.12
	EO LT	y = −0.0055x + 0.003	0.08	0.18	y = 0.0021x + 0.004	0.2	0.05	y = 0.012x−0.007	0.5	<0.001	y = 0.004x−0.002	0.85	<0.001
FW 2	EC	y = 0.002x + 0.0008	0.18	0.07	y = 0.0006x−0.0002	0.5	<0.001	y = 0.0025x + 0.004	0.14	0.11	y = 0.0006x−0.0002	0.5	<0.001
	EC LT	y = 0.0015x + 0.0002	0.41	<0.01	y = 0.0005x + 0.001	0.55	<0.001	y = 0.0011x−0.0004	0.43	<0.01	y = 0.0005x + 0.001	0.55	<0.001
	EO	y = 0.0054x−0.0017	0.35	<0.01	y = 0.0014x−0.0003	0.7	<0.001	y = 0.0099x−0.006	0.43	<0.01	y = 0.0014x−0.0003	0.7	<0.001
	EO LT	y = 0.006x−0.0021	0.5	0.07	y = 0.0014x + 0.001	0.54	<0.001	y = 0.0067x−0.004	0.42	<0.01	y = 0.0014x + 0.001	0.54	<0.001
FW 3	EC	y = 0.0036x−0.0024	0.26	<0.05	y = 0.001x−0.003	0.57	<0.001	y = 0.0053x−0.005	0.45	<0.01	y = 0.001x−0.003	0.57	<0.001
	EC LT	y = 0.0016x + 0.0008	0.3	<0.05	y = 0.0005x + 0.002	0.38	<0.01	y = 0.0015x−0.0006	0.44	<0.01	y = 0.0005x + 0.002	0.38	<0.01
	EO	y = 0.0015x−0.0005	0.35	<0.01	y = 0.0003x + 0.0003	0.46	<0.01	y = 0.0017x−0.0001	0.15	0.1	y = 0.0003x + 0.0003	0.46	<0.01
	EO LT	y = 0.0016x−0.0004	0.5	<0.05	y = 0.0004x + 0.0003	0.63	<0.001	y = 0.0014x−0.0005	0.41	<0.01	y = 0.0004x + 0.0003	0.63	<0.001
FW 4	EC	y = 0.002x−0.002	0.3	<0.05	y = 0.0005x−0.001	0.46	<0.01	y = 0.0036x−0.006	0.6	<0.001	y = 0.0005x−0.001	0.46	<0.01
	EC LT	y = 0.0018x−0.0014	0.72	<0.001	y = 0.0005x + 0.001	0.74	<0.001	y = 0.0025x−0.003	0.5	<0.001	y = 0.0005x + 0.001	0.74	<0.001
	EO	y = 0.0009x−0.0006	0.38	<0.01	y = 0.0002x−0.0003	0.65	<0.001	y = 0.0012x−0.001	0.4	<0.01	y = 0.0002x−0.0003	0.65	<0.001
	EO LT	y = 0.0005x−0.0002	0.85	<0.05	y = 0.0001x + 5*10^−5^	0.78	<0.001	y = 0.0006x−0.0004	0.6	<0.001	y = 0.0001x + 5*10^−5^	0.78	<0.001
FW 5	EC	y = 0.0004x−4*10^−4^	0.5	<0.001	y = 0.00006x + 0.0002	0.37	<0.01	y = 0.0006x−0.0006	0.43	<0.01	y = 0.00006x + 0.0002	0.37	<0.01
	EC LT	y = 0.0005x−0.0006	0.58	<0.001	y = 0.0002x−0.0002	0.76	<0.001	y = 0.0005x−0.0004	0.6	<0.001	y = 0.0002x−0.0002	0.76	<0.001
	EO	y = 0.0006x−0.0006	0.92	<0.001	y = 0.000007x−4*10^−5^	0.4	<0.01	y = 0.0005x−0.0004	0.45	<0.01	y = 0.000007x−4*10^−5^	0.4	<0.01
	EO LT	y = 0.0004x−2*10^−4^	0.75	<0.001	y = 0.00009x−4*10^−5^	0.77	<0.001	y = 0.0001x−7*10^−5^	0.63	<0.001	y = 0.00009x−4*10^−5^	0.77	<0.001
FW 6	EC	y = 0.00009x−0.0001	0.64	<0.001	y = 0.00001x + 2*10^−5^	0.43	<0.01	y = 0.0003x−0.0005	0.56	<0.001	y = 0.00001x + 2*10^−5^	0.43	<0.01
	EC LT	y = 0.0001x−9*10^−5^	0.7	<0.001	y = 0.00003x−1*10^−5^	0.75	<0.001	y = 0.0001x−0.0001	0.76	<0.001	y = 0.00003x−1*10^−5^	0.75	<0.001
	EO	y = 0.00005x−3*10^−5^	0.7	<0.001	y = 0.000007x + 1*10^−5^	0.78	<0.001	y = −0.00009x−7*10^−5^	0.62	<0.001	y = 0.000007x + 1*10^−5^	0.78	<0.001
	EO LT	y = 0.00005x−3*10^−5^	0.74	<0.001	y = 0.00001x + 4*10^−6^	0.54	<0.001	y = 0.00005x−3*10^−5^	0.4	<0.01	y = 0.00001x + 4*10^−6^	0.54	<0.001

### Solid Base of Support

#### Power Spectrum

**Figure 12:**
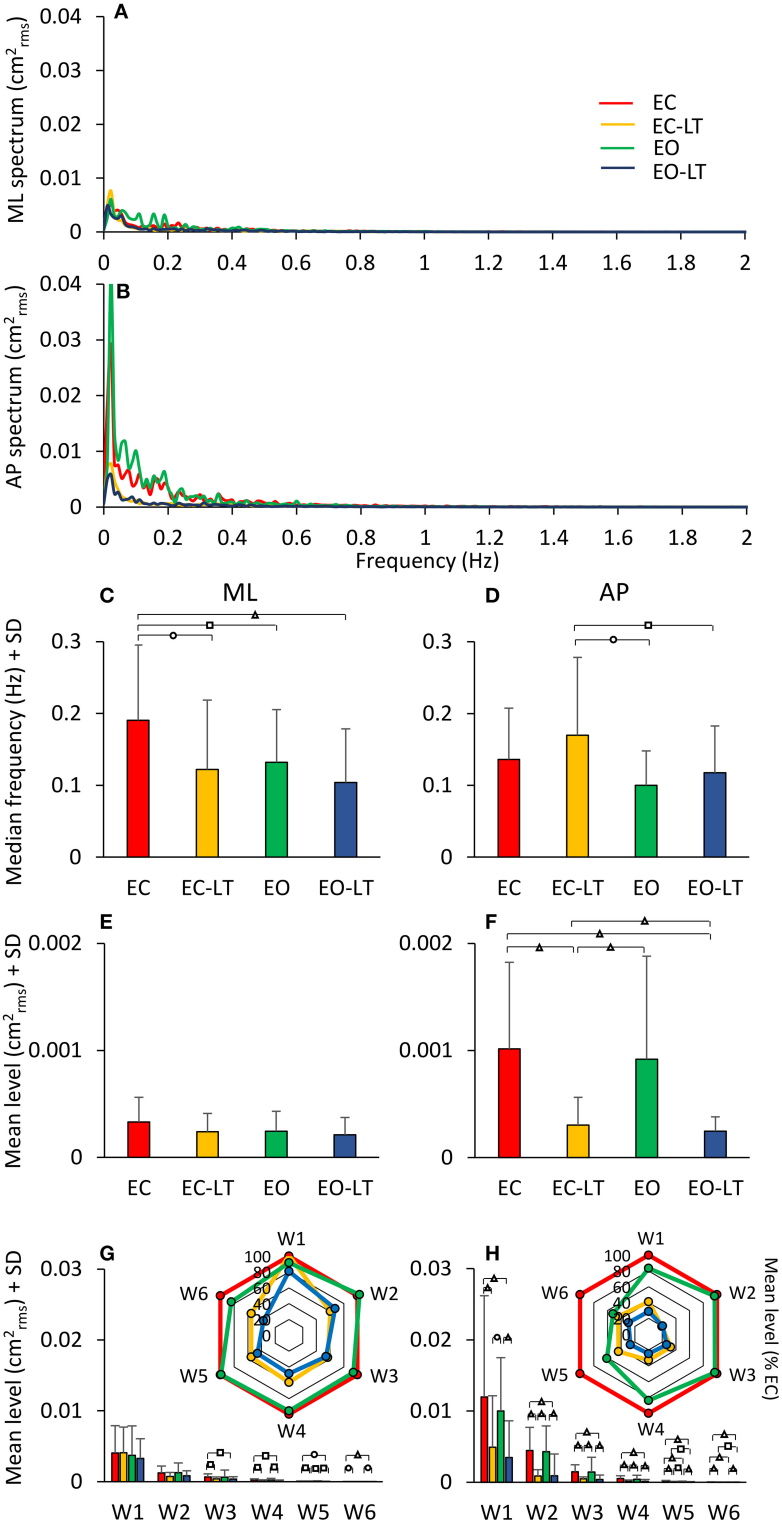
Power spectrum on solid BoS. **(A,B)** compare the power spectra during quiet stance EC (red), EO (green), EC-LT (yellow), and EO-LT (blue). For the low-frequency range, the mean level with EO was not smaller than with EC (for both ML and AP directions). Touch (EC-LT) and vision (EO) produced the smallest amplitudes of the spectrum. The median frequency of the entire spectrum was not much different across conditions **(C,D)**. However, the mean level was larger in AP than ML (both EC and EO), and much larger without touch **(E,F)**. Overall, median frequencies were about half, and the mean level was about half to a quarter of those with foam (compare to Figure 5). The changes with respect to EC of the distinct frequency windows are shown in the radar plots **(G,H)** for both ML and AP directions. Compared to the foam conditions (see Figure 8), the mean levels are less than half. Distinct symbols indicate significant differences (^□^*p* < 0.05; **°***p* < 0.01; ^**Δ**^*p* < 0.001).

The mean power spectra profiles computed for the four tested conditions (EC, EC-LT, EO, EO-LT) when standing on the solid BoS are shown in Figures 12A,B. For each condition and for each subject, the median frequency and the mean level of the spectrum were calculated between 0.01 and 2 Hz (Figures 12C–F).

##### Sensory Conditions

All conditions included, the median frequency was not different between ML and AP [*F*_(1, 18)_ = 0.2, *p* = 0.65]. There was a difference between conditions [*F*_(3, 54)_ = 4.1, *p* < 0.05, *d* = 4.25, $\eta_{\text{p}}^{2}$ = 0.18] and an interaction between median frequency of ML and AP directions and sensory conditions [*F*_(3, 54)_ = 4.01, *p* < 0.05, *d* = 4.14, $\eta_{\text{p}}^{2}$ = 0.18]. For the ML direction, the median frequency was much higher with EC than with all other sensory conditions (*post-hoc, p* < 0.05 for all comparisons). There was no difference between EC-LT and the two conditions with eyes open (EO and EO-LT) (*p* > 0.4 for both comparisons) and between EO and EO-LT (*p* = 0.22). For the AP direction, EC was not different from the other sensory conditions (*p* > 0.1 for all comparisons). EC-LT was larger with respect to EO (*p* < 0.05) and EO-LT (*p* < 0.05). There was no difference between EO and EO-LT conditions (*p* = 0.44).

The mean level of the spectrum between 0.01 and 2 Hz was different between ML and AP [*F*_(1, 18)_ = 16.45, *p* < 0.001, *d* = 1.91, $\eta_{\text{p}}^{2}$ = 0.48]. There was a difference between conditions [*F*_(3, 54)_ = 11.4, *p* < 0.001, *d* = 1.59, $\eta_{\text{p}}^{2}$ = 0.39] and an interaction between ML and AP directions and sensory conditions [*F*_(3, 54)_ = 12.6, *p* < 0.001, *d* = 1.67, $\eta_{\text{p}}^{2}$ = 0.41]. For ML, the mean level was not different between sensory conditions (*post-hoc, p* > 0.2 for all comparisons). For AP, the mean level with EC was higher than with EC-LT and EO-LT (*p* < 0.001), but was not different from EO (*p* = 0.35). The mean level with EC-LT was smaller than EO (*p* < 0.001) and similar to EO-LT (*p* = 0.57). Compared to the foam condition, the median frequency was lower for the solid BoS [*F*_(1, 18)_ = 60.7, *p* < 0.001, *d* = 3.66, $\eta_{\text{p}}^{2}$ = 0.77] when all sensory conditions were included. The mean level of the spectrum between 0.01 and 2 Hz was also different between foam and solid BoS [foam > solid, F_(1, 18)_ = 108.19, *p* < 0.001, *d* = 4.89, $\eta_{\text{p}}^{2}$ = 0.86].

##### Frequency Windows

In Figures 12G,H, the mean level of the spectrum in the different frequency windows and their percent reduction with respect to EC condition is reported for each sensory condition.

There was a difference in the spectrum mean level between the ML and AP directions when all conditions included [*F*_(1, 18)_ = 17.4, *p* < 0.001, *d* = 1.97, $\eta_{\text{p}}^{2}$ = 0.49]. There was also a difference between conditions [*F*_(3, 54)_ = 9.08, *p* < 0.001, *d* = 1.42, $\eta_{\text{p}}^{2}$ = 0.33] and between frequency windows [*F*_(5, 90)_ = 38.1, *p* < 0.001, *d* = 2.91, $\eta_{\text{p}}^{2}$ = 0.68]. There was an interaction between ML and AP directions and sensory conditions [*F*_(3, 54)_ = 10.3, *p* < 0.001, *d* = 1.51, $\eta_{\text{p}}^{2}$ = 0.36], ML and AP directions and frequency windows [*F*_(5, 90)_ = 8.6, *p* < 0.001, *d* = 1.38, $\eta_{\text{p}}^{2}$ = 0.32], between sensory conditions and frequency windows [*F*_(15, 270)_ = 4.8, *p* < 0.001, *d* = 1.03, $\eta_{\text{p}}^{2}$ = 0.21], and between ML and AP directions, sensory conditions and frequency windows [*F*_(15, 270)_ = 4.5, *p* < 0.001, *d* = 0.99, $\eta_{\text{p}}^{2}$ = 0.20]. For the ML direction, there was no significant difference between sensory conditions for W1 and W2 (*post-hoc, p* > 0.2 for all comparisons). In W3 to W6 there was no difference in the mean levels between EC-LT and EO-LT (*p* > 0.6 for all comparisons) and between EC and EO (*p* > 0.3). Touch reduced the level under both EC-LT and EO-LT conditions (*p* < 0.05). The level of the EC-LT condition was not much different to EO (*p* > 0.07), and that of EO-LT was smaller than EO (*p* < 0.05). For the AP direction, touch reduced the mean levels in all the frequency windows (EC-LT vs. EC and EO-LT vs. EO, *p* < 0.001). There was no difference between EC and EO (*p* > 0.07). Similarly, EC-LT and EO-LT were not different across the windows (*p* > 0.3).

#### Geometric Sway Measures

**Figure 13:**
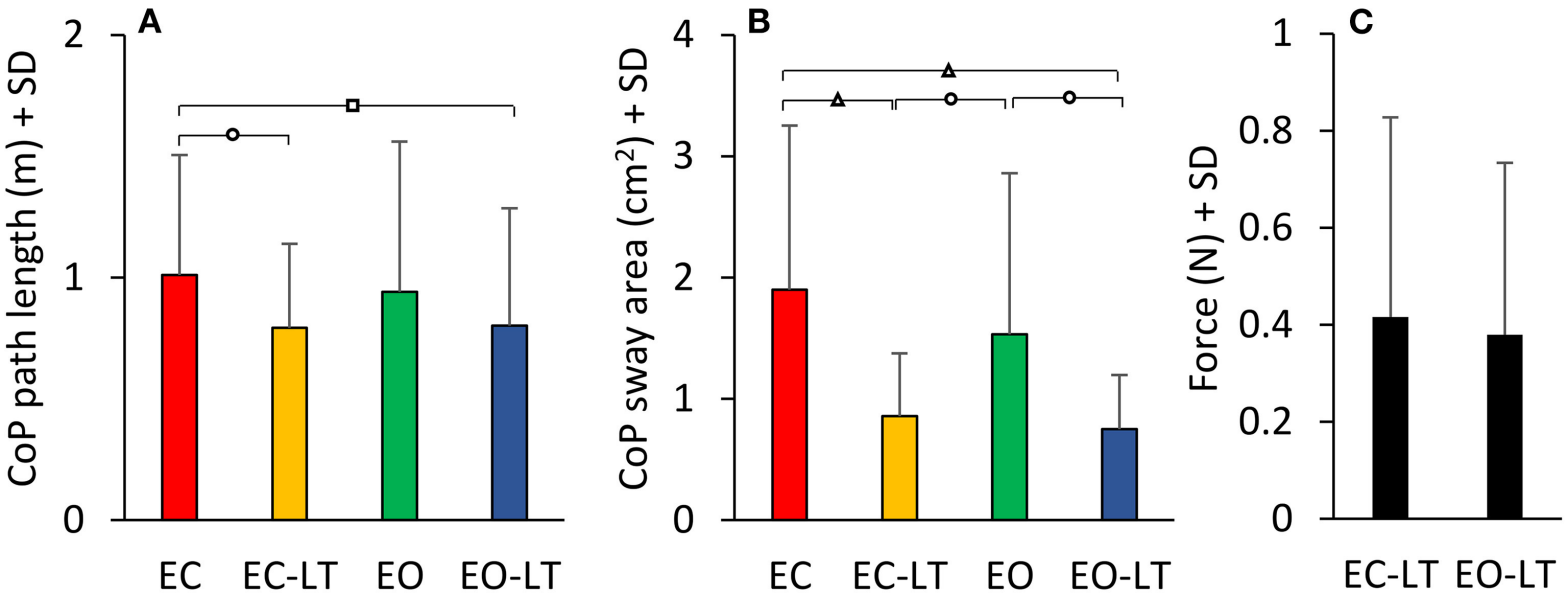
CoP path length and sway area on solid BoS. The path length and 95% ellipse area with the solid BoS are much smaller than in the foam condition. While the path length is not very different across sensory conditions **(A)**, sway area shows larger differences across conditions, the minimal excursions of the CoP being present when touch is available **(B)**. In the solid BoS condition, the touch forces **(C)** are similar, with and without vision. Distinct symbols indicate significant differences (^□^*p* < 0.05; **°***p* < 0.01; ^**Δ**^*p* < 0.001).

The mean CoP path length and the mean sway area calculated across subjects standing on solid BoS are shown in Figures 13A,B. ANOVA on the CoP path length showed a difference between sensory conditions [*F*_(3, 54)_ = 3.46, *p* = 0.02, *d* = 0.88, $\eta_{\text{p}}^{2}$ = 0.16]. With EC, the path length was greater than EC-LT and EO-LT (*post-hoc, p* < 0.05 for both comparisons), but not different from EO (*p* = 0.4). There was no difference between the two conditions with touch (EC-LT vs. EO-LT, *p* = 0.92). ANOVA, on the sway area, showed a significant difference between sensory conditions [*F*_(3, 54)_ = 11, *p* < 0.001, *d* = 1.56, $\eta_{\text{p}}^{2}$ = 0.38]. Sway area with EC and EO was greater than EC-LT (*post-hoc, p* < 0.01 for both comparisons) and EO-LT (*p* < 0.01 for both comparisons). There was no difference in sway area between EC and EO (*p* = 0.17) and between EC-LT and EO-LT (*p* = 0.68). Path length [*F*_(1, 18)_ = 90.5, *p* < 0.001, *d* = 4.48, $\eta_{\text{p}}^{2}$ = 0.83] and sway area [*F*_(1, 18)_ = 93.97, *p* < 0.001, *d* = 4.56, $\eta_{\text{p}}^{2}$ = 0.84] were greater with foam than solid BoS when all sensory conditions are included. Much as with foam, during the trials performed on the solid BoS vision did not affect the force applied by the subjects onto the force pad (Figure 13C) (paired *t*-test, *p* = 0.29). In turn, the touch forces exerted on solid BoS were not different from those recorded when standing on foam [*F*_(1, 18)_ = 2.6, *p* = 0.12].

## Discussion

Our hypothesis was that vision and light touch stabilise body sway through at least partially different actions (31). To this objective, we have computed and analysed both the usual stabilometric indices (sway path and sway area) and the power spectra of the oscillation frequency along with both the frontal and the sagittal planes in healthy young subjects standing on a compliant and on a solid surface.

Spectral analysis of body oscillations during stance has been repeatedly exploited in order to understand the processes underpinning the control of equilibrium in the absence of external perturbations (35–39, 116–120). Singh et al. (98) had a contiguous research question and emphasised open issues in attributing specific frequencies to the effect of different sensory modalities on standing posture. Inconsistencies in the methodological approach across the literature might have detracted researchers and clinicians from the use of standardised approaches. The span of considered frequencies varies across laboratories, and one wonders whether frequencies as high or higher than, say, 2 Hz can have a practical counterpart for the interpretation of the sway of healthy, non-trembling subjects (121, 122). Moreover, the value of the ordinate in the power spectrum is sometimes of difficult interpretation, also because of dissimilar modes in the signal processing (e.g., filtered/non-filtered) and the metrics used in different studies, so that attention seems to have been devoted more to the frequencies themselves than to the effective amplitude of their power spectrum. We have leveraged this approach in order to test the possibility that the effects of different sensory conditions and support bases on the body sway can be easily detected by the frequency analysis compared to the commonly used metrics and can yield details not granted by the simple analysis of the CoP path length or sway area.

We have tried to identify ranges and amplitudes of oscillation frequencies presumably having an actual physical counterpart in the wandering of the CoP of the standing body, and to detect spectrum windows which could be questioned for elucidating the effects of adding haptic and visual sensory information on the CoP oscillations identified in the most unstable condition (eyes closed, EC). As a consequence, we have limited our analysis to the region of the power spectrum (below 2 Hz) encompassing about 99% of the total available spectrum (i.e., containing the frequencies up to 70 Hz, which depend on the frequency of sampling of the CoP signal). We have also checked that the oscillations beyond 2 Hz represent a tiny proportion of the geometric metrics, the sway area, and the path length. The oscillations beyond 2 Hz do indeed represent a minor part of the sway area (<1%) (see Figure 3B). They contribute to the path length by about 50%, though. The incongruous length of the sway path compared to its area is due to the long period of acquisition, when the filtered signal does negligibly oscillate but may show minimal displacements, the sum of which gives rise to sizeable total lengths over the 90 s epoch. Perhaps, for this reason, frequencies from 2 to 20 Hz have been considered in some studies on dizzy patients (123, 124).

With EC foam, the frequency spectra and their amplitudes were broadly similar in both ML and AP directions, but larger in AP for frequencies in the W1 and W2. This was not unexpected because the spontaneously oscillating body during quiet stance does not really care about the space directions along which to move, and the balancing strategies of a double-inverted pendulum are not really functionally separated (56, 125) unless imposed by the feet distance (24, 126, 127). In our subjects, this distance (the outer profile of the feet was about the hip distance) was appropriate for promoting omni-directional sway, as obvious in the shape of the wandering of the CoP on the horizontal plane (see Figures 3A, 10A,C).

The median frequencies are higher without vision (EC). The median frequencies remain high (or get relatively higher in the AP direction) when touch is added to EC. Hence, the median frequency is not a good predictor of the effect of the haptic information. Conversely, vision is associated with low median frequency values. This is true both with and without touch. In general, vision diminishes the median frequency, while touch diminishes the amplitude of the spectrum. It seems that vision prescribes the frequency of oscillation, upon which touch quantitatively modulates the amplitude. As expected, there is a broad correspondence between the amplitude of the spectra and the geometric sway measures (compare Figures 6E,F with Figures 10E,F). Across the sensory conditions, the length of the sway path is broadly reflected in the amplitude of the mean level of the spectrum along the ML direction, and the sway area is rather reflected in the mean level along the AP direction.

### Vision and Touch Stabilise the Standing Body on the Foam

#### Vision

The power spectrum of the oscillation frequencies with EC foam has been considered here the default condition, against which to compare the stabilising effects of vision and touch and both together. Compared to EC, vision (EO) reduced the area of the ellipse by about 61% and the CoP path length by about 53% (see Figure 10). In the spectral analysis, the addition of vision remarkably lowered the median frequency of the entire spectrum. This diminution affected the entire spectrum both in the ML (63%) and in the AP (60%) directions (see Figure 5). This was consistent with a decrease in the amplitude of the medium-high frequencies with sparing of the low frequencies. While the amplitude of each of the oscillation frequency windows was smaller with EO than with EC, the vision had no effect on the spectral mean level of the low-frequency windows in the ML and AP directions. This is in keeping with the findings by Yamagata et al. (86), who showed that slow oscillations, or drifts, appear to be poorly sensitive to vision. Conversely, the reduction was conspicuous for the subsequent windows (between 0.2 and 0.8 Hz), i.e., for oscillation cycles lasting from 5 to nearly 1 s. The highest-frequency windows (beyond 0.8 Hz) were scarcely influenced by vision, much as had been previously shown (56, 117). Under unstable conditions (as standing on an inclined surface or on a balance trainer ball), the slow components of the postural sway appear to depend on vision compared to more stable conditions (128–130). No significant vision effects on the oscillation frequency were noted by Šarabon et al. (82), probably because they measured the average frequency. In our hands, the reduction was clear in the median frequency, just because of the preserved low-frequency and reduced high-frequency oscillations. The frequency window W3 (from 0.2 to 0.44 Hz) should contain frequencies related to ventilation (131), i.e., broadly between 0.2 and 0.3 Hz for ventilation cycles from 12 to 18 per minute, which are probably blunted in the mean spectrum by inter-subject variability. In our hands, the amplitude of the postulated ventilation component diminishes with the general decay of the mean level of the spectrum in the stabilised conditions, making it difficult to draw strong deductions. Interestingly, stabilisation by vision (EO vs. EC) reduced both length and area of the CoP wandering without decreasing the level of the lowest part of the spectrum (the frequency Ws 1 and 2, spanning 0.01–0.2 Hz). The absence of influence of this part of the spectrum on the geometric sway measures is “compensated” by the strong relationships between the level of the higher-frequency windows with CoP path length and sway area (see Figure 11).

#### Touch

A light touch is a potent stabilising stimulus, able to replace vision in subjects with impaired vestibular system (132–134). In the present study, like in several others [(7), see Lackner (47) for a recent review], the haptic information arose from the index fingertip lightly touching the force pad and from the muscles active in this task. In many studies, the vertical force of the fingertip on the force pad representing the earth-fixed reference was generally well-below 1 N and was considered to be inadequate for mechanical stabilisation (60, 135, 136). Importantly, during our experimental trials, the force did not change across the different sensory or BoS conditions.

Compared to EC, the light touch without vision (EC-LT) reduced the sway area (by about 68%) and the path length (40%). In the frequency domain, EC-LT had minimal effects on the median frequencies of the ML and AP spectra (see Figures 6–9). However, EC-LT reduced the level of the spectrum, along with the ML (by 55%) and more so along with the AP (77%) direction (137–139). It is not unlikely that the haptic reference helped diminish the “slow” sway oscillations more in the sagittal than in the frontal plane (140, 141). This would depend on the haptic task, whereby the reference (the fingertip onto the force pad) was located just in front of the subjects, almost coinciding with the sagittal plane, and broadly congruent with the direction of the gaze (142, 143). However, we would not exclude that, when standing on foam, a minor but non-negligible additional advantage might have been furnished by the contact of the finger with the force pad. We have no information on the amplitude or direction of the friction forces on the force pad, though. Although minimal, these cannot be disregarded (144). Since the body oscillations reduction along the ML direction was similar for touch and vision, we suppose that the added reduction in the AP direction was due to the anterior position of the force pad. Qualitative changes occur, however, even when the haptic stimulus is applied to diverse body sites and has no definite direction of action (145). As to the location of the haptic device, we would remind that similar stabilising effects are obtained by using a cane touching the ground (146). The use of a tool is as helpful as the fingertip input and does not produce a different stabilisation.

#### Touch and Vision Stabilise Balance Through Distinct Actions

The stabilising effects of touch (EC-LT) and vision (EO) have been directly compared. While both conditions resulted in a similar reduction of path length and sway area (147), their modulation of the spectrum frequencies was definitely divergent. This was clear in the superimposition of the frequency spectra obtained in the two conditions on foam. While the addition of vision (EO) had no effect on the very low frequencies of the spectrum, the same frequencies abated with touch (EC-LT). Conversely, while the higher frequencies were reduced with vision, a broad peak intruded with touch between 0.3 and 0.8 Hz (148), as if the former frequency window drop and the increase of the latter were a necessary quality of the touch effect. It has been known for decades that touching or even aiming to a stable structure diminishes the amplitude of the leg muscle long-latency reflex responses to stretch (46, 149, 150), whereas the short-latency responses are hardly affected. Touch would not be powerful enough for cancelling the medium-high frequencies, likely sustained by continuous operation of short-chain reflexes that represent a major share of the oscillations EC. Further, a new inter-foot coordination pattern (26) would emerge when a midline reference (the LT in front of the subject) is available and attenuate slow omnidirectional oscillations in favour of fast, short displacements. The sway area reflects this effect, as shown by the major shrinkage of the ellipses fitted to the EC-LT (but not EO) data, with a minor reduction in path length. One might speculate that touch *without* vision sustains the elevated level of excitability of the proprioceptive circuits operating with EC as if this would serve prompt postural corrections when an obstacle challenges the equilibrium in the frontal plane. We would add that, while the spectrum profiles of EC-LT and EO (i.e., during stabilisation by vision or touch) clearly intersected on foam (Figure 8), this pattern disappeared when standing on solid BoS. On the other hand, even the differences between EC and EO disappear with solid BoS, where the sensory information is less crucial for stabilisation.

#### The Combination of Touch and Vision Produces the Maximal Stabilisation

The integration of touch and vision has been often studied in the context of studies on space perception (151). The interaction of both inputs would take place in cortical areas that are related, among other things, with the control of equilibrium (152–155). In our hands, the condition touch and vision together (EO-LT) clearly proved to be able to further reduce the sway area and the path length (more so the area than the path length) compared to EC (area and path length were reduced by 83 and 69%, respectively). Sway area and path length were also reduced compared to touch (EC-LT) (by 47 and 48%) (see Figure 10) and to EO when separately considered (by 57 and 34%), confirming the findings of Honeine et al. (63). Regarding the frequency spectrum, EO-LT was superior in attenuating the oscillations on foam compared to vision alone (EO). However, EO-LT was not superior to touch without vision (EC-LT) in the low-frequency windows (W1 and W2). In this vein, we would mention that, while the integration of haptic and visual inputs in cortical regions likely plays a role, other probably concurrent processes cannot be overlooked. Effectiveness in visuo-haptic integration is enhanced by object-selective brain regions with increased salience of the stimulus (156). Salience can be attributed to our light touch here because attention was devoted to keeping the force within the required range, even if subjects did not look at the force pad with EO. The level of attention would be most likely different in the foam than in the solid BoS condition. In the former case, a precision task would be implicit (141), even if not expressly required. In the latter case, the task can be more easily carried out and provide a simple haptic reference in the absence of accidental displacement (73).

### Foam and Solid BoS

The solid BoS, compared to foam, diminished the median frequency of the spectra almost selectively for the EC condition (both EC and EC-LT), whereas the median frequencies with vision (both EO and EO-LT) were similar in the two BoS conditions. However, the oscillations in the 0.3–0.8 Hz and higher frequency windows (W3–W6) under EC-LT conditions appear only when standing on foam, as if this frequency range were a distinctive feature of standing on a compliant surface. Instead, the mean level of the spectrum was much reduced for all sensory conditions on solid BoS in both ML and AP directions. The effect of touch on the mean level seems to be proportionally stronger on foam in keeping with the observation that unstable balance enhances the haptic sensitivity (157). Generally speaking, it seems that the distinct qualitative contributions to the stabilisation process by vision, touch, or both are hard-wired. These contributions are modulated in amplitude. When the body is in a stable condition (solid BoS), it would continue to operate but are adaptively scaled (or “downweighed”) to the new state.

The balancing behaviour standing on a compliant surface must adapt to the spring-like properties of the foam that influence sway by its own mechanical compliance. This would favour higher activity in the muscles controlling mediolateral oscillations (49, 98). For example, the mean levels of the AP and ML spectra were less different on foam than on solid BoS, suggesting proportionally larger mediolateral adjustments on foam. The inverted pendulum model does not apply to this condition, and movements at several joints contribute to the equilibrium control (13, 158). Analogously, it seems simplistic to posit that the proprioceptive system is disturbed, or its contribution attenuated or invalidated when standing on foam (43, 159, 160). Sway while on foam compared to solid BoS may be less useful for “exploration” of the support base (161) and for “resetting” of the input from the adapting receptors of the foot sole (40). Whereas, pelvis and trunk movements may be more important in getting information about body segments' orientation in space when task difficulty increases (134, 162). The continuous corrections of the body segment displacements on the foam are likely dependent on proprioceptive volleys, which send a continuous (meaningful) input to various regions of the brain and produce appropriate short- and long-latency reflexes (163). Conversely, the vibration of the leg muscles, producing a non-meaningful proprioceptive input, has a smaller effect on body sway while standing on foam (164) or on an unstable support (165, 166). If anything, these findings support the notion that proprioception continues to operate (and likely much more) on a compliant (foam) than solid BoS. The vibration of trunk muscles has, instead, a larger effect compared to the vibration of the leg muscles (164), a finding that we interpret as a shift in excitability of different circuits rather than mere disruption of proprioceptive information by vibration. Of note, touch increases the postural tone in trunk muscles (167). It is not clear, though, whether the new trunk postural activity can be responsible for the relative increase in medium-high frequencies seen while standing on foam in EC-LT condition (168, 169).

With EC, our subjects were actually unstable on foam (but never made a step during these trials) and became stable with touch (EC-LT). Touch devoid of mechanical action (always <1 N) could have had such effect just because proprioception was properly working. In a sense, the ampler the joint movements on foam, the more substantial the proprioceptive input from multiple muscles, not excluding those of the forearm muscles enabling and contributing to a significant haptic input (64, 170). Mastering a complex proprioceptive input can be difficult and lead to instability. However, there must be a large safety margin compensating for unpredictable alteration in proprioceptive input. Young subjects, healthy except for the Marfan syndrome, a disorder targeting the connective tissue, show impaired balance control under critical conditions (unstable BoS, eyes closed) (171). However, just a few of them had to be supported during the trials despite presumably altered reflex patterns due to their unique joint hypermobility.

### Frontal and Sagittal Planes

Control of balance in the mediolateral direction is critical (52, 172) and is often impaired in older adults and when the asymmetry of stance is present, like for instance in stroke patients (173, 174). There were considerable differences in the mean level of the spectrum of all the frequency windows between ML and AP directions, and the overall pattern of the effects of touch and vision were distinct along the frontal and sagittal planes and both on foam and solid BoS (compare Figures 8, 12). On the other hand, the median frequency was not different between the frontal and the sagittal planes for both foam and solid BoS. Touch compared to no-touch, regardless of vision availability, exerted a larger stabilising effect in the AP than in the ML direction. Such a stabilised condition (EC-LT and EO-LT) may have reduced the “rambling” component of the control while favouring the “trembling” omponent (86, 175), thanks to the haptic reference in the AP direction. With vision, a difference also emerged between ML and AP directions in the mean level of the spectrum because spectrum amplitude relatively prevailed in the AP direction on solid BoS.

### Limitations

The sample size was not determined prior to the study. However, the effect size of the significant differences indicates always large effects. Given our sample size of 19 participants, the study proved to have a sufficient power (>80%) to detect an effect in median frequency larger than 0.12 Hz between the EC-LT and EO conditions. The data have been collected here and analysed on the basis of one single trial per subject per condition (i.e., the first trial of a series of eight trials), administered in order to investigate the effect of the sensory conditions in the adaptation to repeated stance performances. Given the inter-individual variability in the stance performance (9, 176), particularly when standing on foam, this procedure is certainly a limitation however hardly avoidable because repetition of stance trials produces significant adaptation in the balancing pattern (8, 127, 177–180). Alsubaie et al. (181) have recently shown that different measures of postural sway are reliable when recorded at two visits 1 week apart, including measures with unstable BoS and sensory conditions. We do not know whether the frequencies might change as a function of the viewing scene (the characteristics of the patterned environment or the visual target being close or far) or of the position, texture, and orientation with respect to the body of the force pad, or of the force exerted by the finger. Further, the effect of the inner mechanical spring-like properties of the foam on the recorded signal has not been addressed, thereby preventing quantitative considerations on the role of proprioception standing on foam (or on solid BoS as well) and its potential interaction with vision and touch. Moreover, the feet position has not been manipulated, contrary to Šarabon et al. (82), who however found no major difference in the oscillation frequencies across different positions.

No estimation of the role of the vestibular information is provided here (182, 183), and no measure of the displacement in space of body segments such as the head and the pelvis. The information from the plantar foot sole, certainly different between foam and solid BoS, must have played a role. It has been proposed that our perception of verticality on a compliant surface, dependent in part on the plantar foot mechanoreceptor input, decreases when vision is not available (39, 184). We made no attempt to assess the contribution of these receptors. Electrical activity of the muscles involved in the control of equilibrium under the tested conditions has not been recorded, either. This information would help define the coordination properties underpinning the changes in oscillation frequency prescribed by the sensory conditions and the factors leading to the specific power spectrum features of postural sway. Moreover, this preliminary investigation is limited to young adult healthy subjects, and no information on the behaviour of aged healthy persons (185–187) or of patients with equilibrium disorders has been collected and analysed in these conditions according to this methodology (188).

## Conclusion

The data suggest that the oscillation frequency analysis, in spite of its relative complexity, gives information on the control mode of critical stance exerted by different sensory inputs, not supplied by common and simpler geometric sway measures. The use of foam highlighted a significant increase in medium-high frequencies with touch in the absence of vision compared to vision alone. In perspective, the approach based on the analysis of distinct windows in the frequency spectrum of the body oscillations would help postulate the existence, and define the respective mechanisms, of the specific process through which different sensory information contributes to body stabilisation under critical conditions (31). Differences in balance control between young and older subjects would also be easily defined and exploited as a measure of balance alterations in patients due to impairments of various origins (189–192).

## Data Availability Statement

The original contributions presented in the study are included in the article. Further inquiries can be directed to the authors upon reasonable request.

## Ethics Statement

The studies involving human participants were reviewed and approved by the Comitato Tecnico Scientifico Ethics Board at the ICS Maugeri SB (Institute of Care and Research, IRRCS). All subjects provided written informed consent to participate in this study.

## Author Contributions

MS conceived the idea for the manuscript. SS performed the recruitment of participants and the collection of data. SS and MS performed the data analysis and drafted the article. AN revised it critically for important intellectual content. All authors approved the submitted version.

## Funding

The funding for this study was provided by the Ricerca Corrente of the Italian Ministry of Health.

## Conflict of Interest

The authors declare that the research was conducted in the absence of any commercial or financial relationships that could be construed as a potential conflict of interest.

## Publisher's Note

All claims expressed in this article are solely those of the authors and do not necessarily represent those of their affiliated organizations, or those of the publisher, the editors and the reviewers. Any product that may be evaluated in this article, or claim that may be made by its manufacturer, is not guaranteed or endorsed by the publisher.
